# DDX56 modulates post-transcriptional Wnt signaling through miRNAs and is associated with early recurrence in squamous cell lung carcinoma

**DOI:** 10.1186/s12943-021-01403-w

**Published:** 2021-08-26

**Authors:** Qingqing Wu, Xiaoyang Luo, Mikkel G. Terp, Qingrun Li, Yuan Li, Lei Shen, Ying Chen, Kirstine Jacobsen, Trever G. Bivona, Haiquan Chen, Rong Zeng, Henrik J. Ditzel

**Affiliations:** 1 Shanghai Institute of Biochemistry and Cell Biology, Center for Excellence in Molecular Cell Science, Chinese Academy of Sciences, Shanghai, 200031 China; 2 Department of Cancer and Inflammation Research, Institute of Molecular Medicine, University of Southern Denmark, J.B. Winsløwsvej 25, 5000 Odense C, Denmark; 3 Department of Thoracic Surgery, Fudan University Shanghai Cancer Center, Shanghai, 200032 China; 4 Department of Oncology, Fudan University Shanghai Medical College, Shanghai, 200032 China; 5 Department of Pathology, Fudan University Shanghai Cancer Center, Shanghai, 200032 China; 6 Diller Family Comprehensive Cancer Center, University of California, San Francisco, CA USA; 7 Department of Oncology, Odense University Hospital, 5000 Odense, Denmark; 8 Department of Clinical Research, University of Southern Denmark, 5000 Odense, Denmark; 9 Academy of Geriatric Cancer Research (AgeCare), Odense University Hospital, 5000 Odense, Denmark

**Keywords:** DDX56, Squamous cell lung cancer, Wnt signaling pathway, miRNA

## Abstract

**Background:**

Early recurrence is a major obstacle to prolonged postoperative survival in squamous cell lung carcinoma (SqCLC). The molecular mechanisms underlying early SqCLC recurrence remain unclear, and effective prognostic biomarkers for predicting early recurrence are needed.

**Methods:**

We analyzed primary tumor samples of 20 SqCLC patients using quantitative proteomics to identify differentially-expressed proteins in patients who experienced early versus late disease recurrence. The expression and prognostic significance of DDX56 was evaluated using a SqCLC tumor tissue microarray and further verified using different online databases. We performed in vitro and in vivo experiments to obtain detailed molecular insight into the functional role of DDX56 in SqCLC.

**Results:**

We found that DDX56 exhibited increased expression in tumors of patients who experienced early versus late disease recurrence. Increased DDX56 expression in SqCLC tumors was subsequently confirmed as an independent prognostic factor of poor recurrence-free survival in independent SqCLC cohorts. Functionally, DDX56 promotes SqCLC cell growth and migration in vitro, and xenograft tumor progression in vivo. Mechanistically, DDX56 post-transcriptionally promotes expression of multiple Wnt signaling pathway-related genes, including CTNNB1, WNT2B, and represses a subset of miRNAs, including miR-378a-3p, a known suppressor of Wnt signaling. Detailed analysis revealed that DDX56 facilitated degradation of primary miR-378a, leading to down-regulation of mature miR-378a-3p and thus derepression of the target gene WNT2B.

**Conclusion:**

We identified DDX56 as a novel independent prognostic biomarker that exerts its oncogenic effects through miRNA-mediated post-transcriptional regulation of Wnt signaling genes to promote early SqCLC recurrence. DDX56 may assist in identifying SqCLC patients at increased risk of early recurrence and who could benefit from Wnt signaling-targeted therapies.

**Supplementary Information:**

The online version contains supplementary material available at 10.1186/s12943-021-01403-w.

## Background

Lung cancer is the leading cause of cancer-related death worldwide at almost 1.4 million deaths per year [1]. Nearly 85% of lung cancers are non-small-cell lung cancers (NSCLC) [2], and of these, squamous cell lung cancer (SqCLC) comprises about 30% [3]. Early stage NSCLC is primarily treated by surgical resection [4]. Even after complete resection, 30% to 55% of NSCLC patients develop recurrence and die of their disease [5], the 5-year survival rate for patients with resected SqCLC being only 48–59% [6]. Therefore, postoperative recurrence is a major obstacle to long-term survival in SqCLC patients.

The Wnt signaling pathway plays a critical role in maintaining stemness of regular stem cells and cancer stem cells (CSC), and has been linked to recurrence of various cancers [7–10]. Recent studies in hepatocellular carcinoma (HCC) showed that increased AKIP1 and PRC1 expression leads to activation of Wnt signaling, which promotes early HCC recurrence [9, 11]. A study in basal cell carcinoma (BCC) demonstrated that Lgr5-positive BCC cells with active Wnt signaling are slow-cycling and mediate BCC relapse after therapy [12]. The importance of Wnt signaling in recurrence was also demonstrated in glioblastoma, where epigenetic activation of WNT5A expression was shown to contribute to tumor recurrence by promoting differentiation of glioblastoma stem cells into endothelial cells [13]. Nevertheless, the detailed molecular mechanism underlying cancer early recurrence, particularly in regulating Wnt signaling in SqCLC, remains unclear. Moreover, current TNM staging is not sufficient to accurately predict recurrence and postoperative prognosis for these patients. To refine the risk of progression and assess the outcome for SqCLC, some prognostic markers have been proposed, such as a gene expression signature consisting of the three genes CSF1, EGFR and CA IX [14, 15] and the protein cathepsin B [16]. However, few of these markers have been shown to be independent of clinical and pathologic features or linked to postsurgical recurrence of SqCLC. Therefore, optimal prognostic markers that can refine prognostic estimates beyond TNM stage and predict early recurrence following resection of SqCLC are critically required.

The DEAD-box family is the largest family of RNA helicases, with 37 members in humans [17], and is involved in nearly all aspects of RNA metabolism and cellular processes requiring manipulation of the RNA structure, such as transcription, pre-mRNA splicing, translation, miRNA processing and mRNA decay [18, 19]. Recent studies showed that overexpression of DEAD box proteins has frequently been linked to poor prognosis in cancer, e.g., increased expression of DDX1 mRNA has been shown to be an independent prognostic marker for early recurrence in breast cancer [20]. Furthermore, high DDX3 levels have been reported to correlate with poor prognoses in gallbladder and breast cancer [21, 22]. Finally, DDX39 has been shown to be a prognostic marker for gastrointestinal stromal tumors associated with metastasis and poor clinical outcomes [23]. In contrast, little is known about the function of DDX56 other than its involvement in ribosome synthesis and assembly of infectious West Nile virus particles [24–26]. Only very recently has it been shown that DDX56 expression is associated with lymphatic invasion and distant metastasis in colorectal cancer [27], and frequently upregulated in tumor tissues and cell lines of osteosarcoma [28], suggesting a potential oncogenic role of DDX56 in cancers.

We investigated differentially expressed protein in resected primary SqCLC tumor tissues from patients who experienced early (ER) versus late disease recurrence (LR) after surgery using iTRAQ-based quantitative proteomics. Among the top significantly upregulated proteins in ER vs. LR SqCLC tumors, DDX56 was identified and further shown to be an independent prognostic marker of poor recurrence-free survival (RFS) in an independent validation cohort of SqCLC patients. Analysis of the functional role of DDX56 in SqCLC cell lines and mouse models showed that DDX56 stimulates growth and migration of SqCLC cells in vitro, progression of xenograft tumors in vivo, and promotes expression of the Wnt signaling pathway-related genes, including WNT2B, through repressing a subset of miRNAs.

## Materials and methods

### Patient samples

Two cohorts of SqCLC patients from Fudan University Shanghai Cancer Hospital were invited to participate. Cohort 1 consisted of 20 patients who underwent surgery between January 2007 and December 2009 and who were part of a previously described cohort of 106 SqCLC patients [29]. Surgically-resected tumor tissues and adjacent normal tissues from cohort 1 were collected and immediately stored in liquid nitrogen, and the tumor tissues were used for proteomic analysis. Clinical data on the cohort is detailed in Additional file 1: Table S1. Cohort 2 consisted of 56 patients who underwent surgery between December 2010 and July 2011, and whose tumors were used to construct tissue microarray for immunohistochemical (IHC) analysis. Inclusion criteria were confirmed diagnosis of SqCLC, no distant metastasis or other surgical contraindications, and the ability to provide written informed consent. Patients with other malignancies or with chronic or acute inflammatory conditions were excluded. None of the patients had received neoadjuvant treatment prior to surgery. The patients in cohort 1 who were allocated to the early disease recurrence (ER) group experienced recurrence within 10 months after surgery, while patients allocated to the late disease recurrence (LR) group experienced recurrence > 30 months after surgery. In the proteomic analysis, each ER sample was paired with an LR sample exhibiting similar clinical characteristics, including gender, smoking history, age and pathological characteristics, including TNM stage. The study was approved by the ethical committee of Fudan University Shanghai Cancer Center. Informed consent was obtained from all participants.

### Proteomic analysis

iTRAQ (isobaric tagging for relative and absolute quantification)-based quantitative proteomics was performed to determine the differential expression of proteins between the two patient tumor groups. The iTRAQ labeled sample mixtures were fractionated on Agilent 1100 HPLC using SCX column, then were analyzed using an LTQ Orbitrap velos mass spectrometer (Thermo Fisher Scientific) coupled with an UltiMate 3000 Nano LC Systems (Dionex, Thermo Fisher Scientific). Further details are available in Additional file 1: Supplementary Methods. The mass spectrometry proteomics data that support the findings of this study have been deposited to the ProteomeXchange Consortium via the PRIDE partner repository with the dataset identifier PXD009383.

### Tissue microarray (TMA) and immunohistochemistry (IHC)

Tissue microarrays (TMAs) containing 56 cores of SqCLC tissues and 4 cores of adjacent normal lung tissues of 2 mm diameter were constructed from formalin-fixed, paraffin-embedded (FFPE) tissue blocks according to previously described methods [30]. IHC for DDX56 protein was performed on the TMA slide using a commercial anti-DDX56 antibody (1:200, sc-393078, Santa Cruz, CA, USA). For detailed IHC methods, please refer Additional file 1: Supplementary Methods. Among the 60 cores, 6 were lost during processing. The staining was graded by a highly experienced pathologist blinded to all clinicopathologic data, as – (negative), + (weak staining), +  + (moderate staining), and +  +  + (strong staining). A total of 52 cores were scored and are shown in Additional file 2: Table S2 with clinical details of the patients.

### Cell culture, transfection and plasmid constructs

The human SqCLC cell line H226 and SK-MES-1 were purchased from ATCC and the Cell Bank of Type Culture Collection of Chinese Academy of Sciences (CBTCCCAS), respectively. DDX56 siRNA #1 (SI00361781), DDX56 siRNA #2 (SI04238157), and negative control siRNA (SI03650318) were purchased from Qiagen (Hilden, Germany). DGCR8 siRNA and negative control siRNA were purchased from Genepharma (Shanghai, China). Stable knockdown of DDX56 in H226 or SK-MES-1 cells was performed by using MISSION shRNA lentiviral transduction particles targeting DDX56 (SHCLNV-NM_019082, Sigma-Aldrich) according to the manufacturer’s instruction. MISSION pLKO.1-puro non-target shRNA control transduction particles (SHC016V, Sigma-Aldrich) were used to generate a control H226 or SK-MES-1 cell line. miR-378i mimic, miR-378a-3p mimic, miRNA mimic negative control, miRNA-378a-3p inhibitor and miRNA inhibitor negative control were purchased from Genepharma. Full-length cDNA (CCDS ID: CCDS5492.1) of the DDX56 transcript variant 1 (NM_019082.4) was cloned from H226 cells. The cDNA was inserted into a pCMC-3Tag-3A vector (Agilent Technologies) to construct the pCMV-FLAG-DDX56 expression vector that encoded a fusion DDX56 protein with 3 × FLAG tag at its C-terminus. The inserted sequence was confirmed by DNA sequencing. For detailed cell culture and transfection methods, please refer to Additional file 1: Supplementary Methods.

### Cell growth, transwell cell migration, wound healing, and apoptosis assays

For the cell growth assay, cells transfected with DDX56-specific siRNAs, control siRNAs, pCMV-DDX56 or empty pCMV vector were seeded into 24-well plates (4 × 10^4^ per well) and, 24, 48, 72 and 96 h after transfection, cells were stained with 0.5% crystal violet stain solution. For miRNA mimic experiments, cells transfected with miR-378a-3p, miR-378i and negative control mimic (4 × 10^4^ per well) were seeded into 24-well plates, and stained with 0.5% crystal violet stain solution 24, 48, 72 and 96 h after transfection. Cell growth was evaluated using the crystal violet staining. For the transwell cell migration assay, H226 and SK-MES-1 cells were transfected with DDX56-specific siRNAs or control siRNA, after which cell migration was assessed using Transwell chambers (8.0-um pore size; Corning, New York, NY). For the wound healing assay, H226 cells were transfected with DDX56-specific siRNAs or control siRNA, after which cell migration was assessed by the ability of the cells to migrate into a cell-free area. For the apoptosis assay, a cell death detection ELISA kit (Roche) was used to investigate the effects of DDX56 reduction in H226 cells on serum deprivation-induced apoptosis. For detailed methods, please refer to Additional file 1: Supplementary Methods.

### qRT-PCR and capture of nascent RNAs

Total RNA was extracted from cells using TRIzol reagent (Invitrogen). For quantitative analysis of mRNAs or primary miRNAs, cDNA synthesis was carried out using 500 ng of total RNA and the iScriptc DNA Synthesis Kit (Bio-Rad, Hercules, CA, USA) according to manufacturer's instructions. qRT-PCR was performed using iQ SYBR Green supermix (Bio-Rad) using the CFX96 real-time PCR Detection System (Bio-Rad). Relative gene expression levels of mRNAs or primary miRNAs were normalized using the reference gene GAPDH. For quantitative analysis of mature miRNAs, cDNA synthesis was carried out using 1 μg of total RNA and the miScript II RT kit (Qiagen) according to manufacturer's instructions. qRT-PCR was performed using miScript PCR Starter Kit (Qiagen) using the CFX96 real-time PCR Detection System (Bio-Rad). Relative gene expression levels of miRNAs were normalized using the reference gene U6B. All primer sequences are listed in Additional file 1: Table S3. The Click-it Nascent RNA Capture Kit (Invitrogen) was used to capture the nascent RNAs. For the detailed method, please refer to Additional file 1: Supplementary Methods.

### Western blotting

The extracted proteins from tissue or cell samples were separated on 12% SDS-PAGE and electroblotted onto PVDF membranes, which were then blocked in TBST buffer containing 5% BSA for 1 h at room temperature and incubated with primary antibodies. The membranes were then washed with TBST and incubated with HRP-conjugated secondary antibody (Santa Cruz) in TBST buffer for 2 h at room temperature, followed by washing in TBST buffer prior to visualization of immunoreactive bands using an ECL Prime Western Blot kit (GE Healthcare) and ImageQuant LAS4000(Fujifilm). Primary antibodies included DDX56, β-Catenin, Wnt-2b, HSP90, FLAG, DGCR8, GAPDH and β-actin. Further details about the Western blotting method and antibodies used are available in Additional file 1: Supplementary Methods.

### mRNA and miRNA microarray gene expression profiling

H226 cells transfected with DDX56 siRNA and control siRNA were incubated for 48 h. For mRNA expression profiling, total RNA was isolated from cells using Trizol reagent (Invitrogen) and arrayed using Affymetrix Human U133 Plus 2.0 Array. For miRNA expression profiling, miRNA was isolated from cells using miRNeasy mini kit (Qiagen) and arrayed using the Agilent Human miRNA (8 × 60 K) Microarray (Agilent Technologies). Further detailed information of mRNA and miRNA microarray are available in Additional file 1: Supplementary Methods. mRNA microarray and miRNA microarray data that support the findings of this study have been deposited in the ArrayExpress database at EMBL-EBI (www.ebi.ac.uk/arrayexpress) under accession number E-MTAB-6675 and E-MTAB-6676, respectively.

### Cell line derived tumor xenograft mouse models

H226 cells stably transduced with DDX56 shRNAs or control shRNAs were harvested, washed in phosphate buffered saline (PBS) and resuspended in extracellular matrix from the Engelbreth-Holm-Swarm sarcoma (Sigma-Aldrich). Tumor cells (1 × 10^6^) were subsequently injected subcutaneously in the right flank of eight-week-old female NOG CIEA mice (Taconic). At study endpoint (week 5), the mice were euthanized by cervical dislocation and tumors were formalin-fixed and paraffin-embedded. All animal experiments were performed at the Animal Core Facility at University of Southern Denmark and approved by The Experimental Animal Committee, The Danish Ministry of Justice.

### RNA stability assay

H226 cells were transfected with DDX56 siRNA, control siRNA, pCMV-DDX56 and pCMV empty vector. Cells were treated with 20 μg/mL actinomycin D 48 h post-transfection, and total RNA was extracted at 0, 3 and 6 h thereafter. Levels of primary miR378a and 18 s rRNA were determined by qRT-PCR.

### Statistics and bioinformatics analysis

Functional ontology enrichment analysis and KEGG pathway enrichment analysis were performed using DAVID bioinformatics resources 6.7 (http://david.abcc.ncifcrf.gov/). Statistically overrepresented GO terms were identified among genes significantly up- or down-regulated in ER patients compared to LR patients. The biomarker validation tool KMplot (http://kmplot.com/analysis/) with published gene expression datasets were used to validate the prognostic effect of candidate proteins. The patient samples in datasets were split into two groups according to auto-selected cutoff. The overall survival (OS) and RFS of the two patient groups was compared by Kaplan–Meier survival plot, Hazard ratio (HR) and two-sided *P* value (*P* < 0.05) calculated using log-rank testing. Univariate and multivariate cox regression analysis were performed using R packages: survival and survminer, clinicopathologic factors (Wald test, *p* < 0.05) analyzed by univariate analysis were selected for multivariate analysis. Kaplan–Meier survival curves were generated using R packages: survival and KMsurv. R (version 4.0.0) and RStudio (version 1.1.463) were used for the statistical analysis. miRNA gene target prediction was performed using MiRNA Targets Prediction (v2.0 beta) http://shbio.cn/analysis.html based on data from 5 databases including TARGETMINER, miRDB, microRNA, TarBase and RNA22. Prediction of miRNA target sites was performed by TargetScan (v7.1; www.targetscan.org) [31].

## Results

### Identification of differentially-expressed proteins associated with time to recurrence of resected SqCLC

**Fig. 1:**
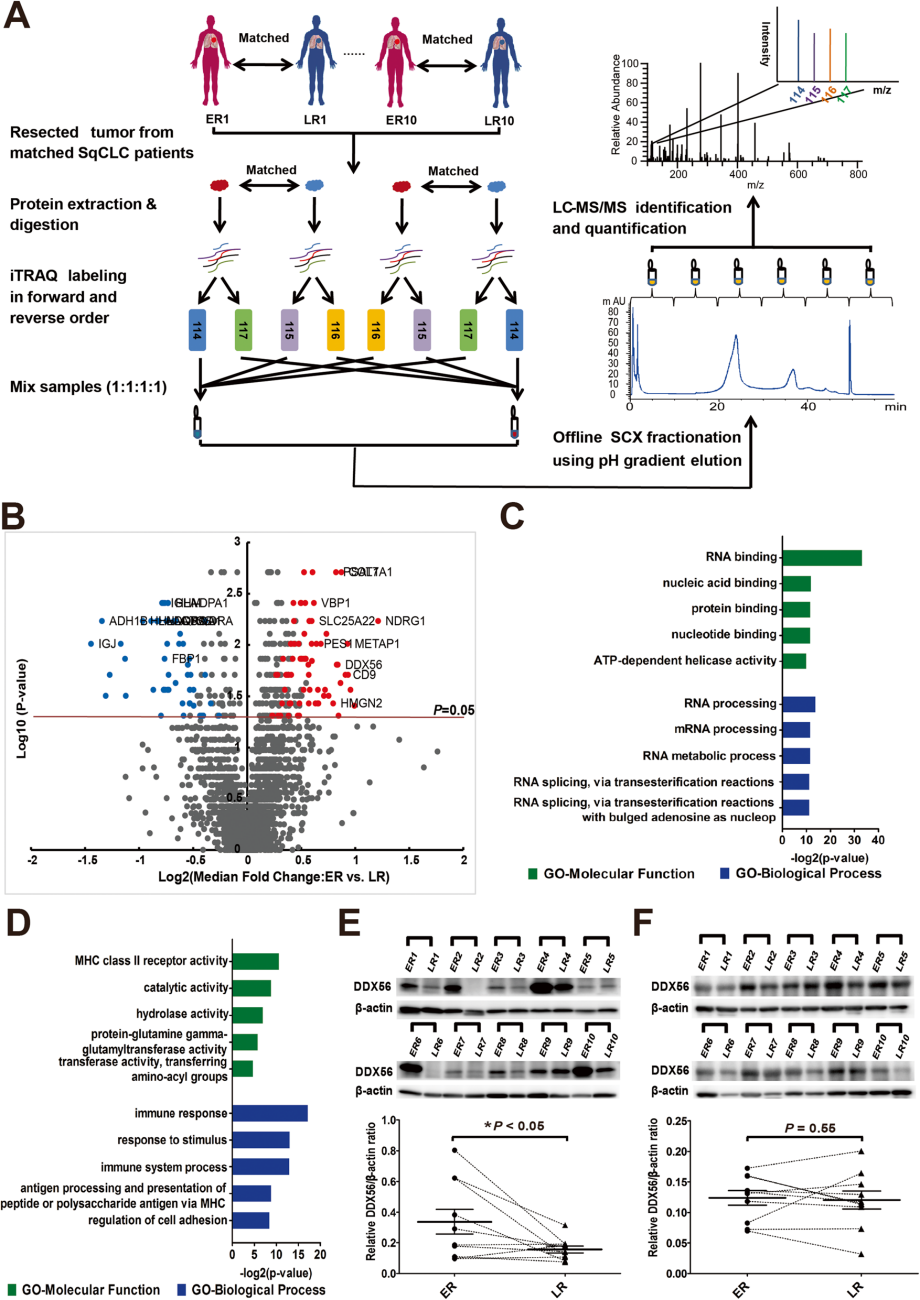
Identification of differentially-expressed proteins in primary squamous cell lung cancer (SqCLC) tissues of patients exhibiting early recurrence (ER) versus late recurrence (LR). **a** Experimental scheme of quantitative proteomic analysis. SqCLC tumor sample of patient with early recurrence (within 10 months) following surgery was paired with a SqCLC tumor sample of a patient with late recurrence (> 30 months) based on similar clinical and pathological features. After tissue lysis and tryptic digestion, each pair of peptide samples were labeled with iTRAQ reagents twice in two different orders (forward and reverse), and were mixed in a ratio of 1:1:1:1. Each mixed iTRAQ-labeled proteomic sample was fractionated into 6 fractions by SCX chromatography using a pH gradient elution, and then analyzed by LC–MS/MS. **b** Volcano plot constructed using median fold-change values and p-values to compare protein expression changes between ER and matched LR patients. Red dots represent significantly upregulated proteins and blue dots represent downregulated proteins (> 1.5-fold change in at least 4 sample sets and *P* < 0.05). The horizontal line represents a *P* value cut off < 0.05. *P* values were calculated using two-sided one sample Wilcoxon signed rank test. **c** and **d** Gene ontology (GO) classification of the 71 upregulated genes (**c**) and 60 downregulated genes (**d**) identified by proteomic analysis. Top 5 enriched GO terms of each category are shown. Green bars represent molecular function terms; blue bars represent biological process terms. The horizontal axis represents the –log2 (p-value). *P* < 0.05 was used as the threshold for selecting significant GO categories. **e** and **f** Western blots were performed to confirm altered DDX56 protein levels in tumor tissues as identified by mass spectrometry analysis, and to further analyze DDX56 protein levels in corresponding adjacent normal lung tissues from the same ER and matched LR patients. **e** Western blot analysis of DDX56 in tumor tissues from paired ER or LR SqCLC patients in cohort 1. Band intensity of DDX56 for the paired ER or LR tumor tissue samples is shown as quantified by densitometry and normalized to β-actin. **f** Western blot analyses of DDX56 in adjacent normal tissues from patients in cohort 1. Band intensity of DDX56 for the paired ER or LR patient adjacent normal tissue samples as shown in **e** quantified by densitometry and normalized to β-actin. Horizontal bars represent the median value with standard error of the mean. *P* value is determined by two-sided paired Wilcoxon signed-rank test. * *P* < 0.05

To identify proteins exhibiting altered expression associated with time to recurrence of SqCLC, we compared the proteome of primary SqCLC tumor tissue from patients with early recurrence (ER, < 10 months) versus late recurrence (LR, > 30 months) following primary surgery. iTRAQ-based quantitative 2D LC–MS/MS proteomics were performed on 20 SqCLC tumor samples (10 ER, 10 LR) using a matched pair design in which each ER tumor sample was paired and analyzed together with a LR tumor sample matched based on similar patient clinicopathologic features, including gender, smoking history, age and TNM status. The experimental scheme for quantitative proteomic analysis is shown in Fig. 1 a. From the MS data, a total of 3209 unique protein groups were identified (FDR < 0.01, identified by at least 1 unique peptide), and of these, 2402 protein groups were identified in at least 6 sample pairs and used in further statistical analysis. Of these, 131 proteins (71 up- and 60 down-regulated) were found to exhibit significantly altered expression in ER versus LR patients (> 1.5-fold change in at least 4 sample sets and p-value < 0.05, two-sided one sample Wilcoxon signed rank test) including DDX56, a member of the DEAD-Box Helicase (DDX) family (Fig. 1 b, Additional file 3: Table S4).

To investigate the biological functions of the significantly regulated proteins, we performed Gene Ontology (GO) analysis using DAVID Bioinformatics Resources 6.7. Interestingly, the GO analysis revealed that the downregulated proteins in ER versus LR in SqCLC tumors were primarily related to immune response, while the upregulated proteins were mainly involved in RNA processing (Fig. 1 c and d). The top 10 up- and top 10 downregulated proteins (Additional file 1: Table S5) were then manually evaluated based on literature and public databases. Most of the top-10 upregulated proteins had already been reported to play a role in oncogenesis and/or to be positively associated with poor prognosis in various cancers, including NDRG1, PSAT1, COL7A1, SLC25A22 and CD9, classified as cancer-related or disease-related genes in the Human Protein Atlas (http://www.proteinatlas.org/). In contrast, the function of DDX56, a member of DEAD-box RNA helicase family, has received little attention and is completely unexplored in SqCLC.

To validate the altered DDX56 protein level as identified by mass spectrometry analysis, we analyzed the DDX56 expression in the same matched tumor tissues from ER and LR SqCLC patients using Western blotting. In agreement with the mass spectrometry data, significantly higher protein levels of DDX56 were observed in ER vs LR SqCLC patients (Fig. 1 e). We further analyzed DDX56 protein levels in corresponding adjacent normal lung tissues from the same SqCLC patients to investigate whether altered levels were also observed between ER and matched LR patients (Fig. 1 f), but no significant difference in DDX56 levels was observed.

### High expression of DDX56 is associated with poor prognosis in SqCLC patients

**Fig. 2:**
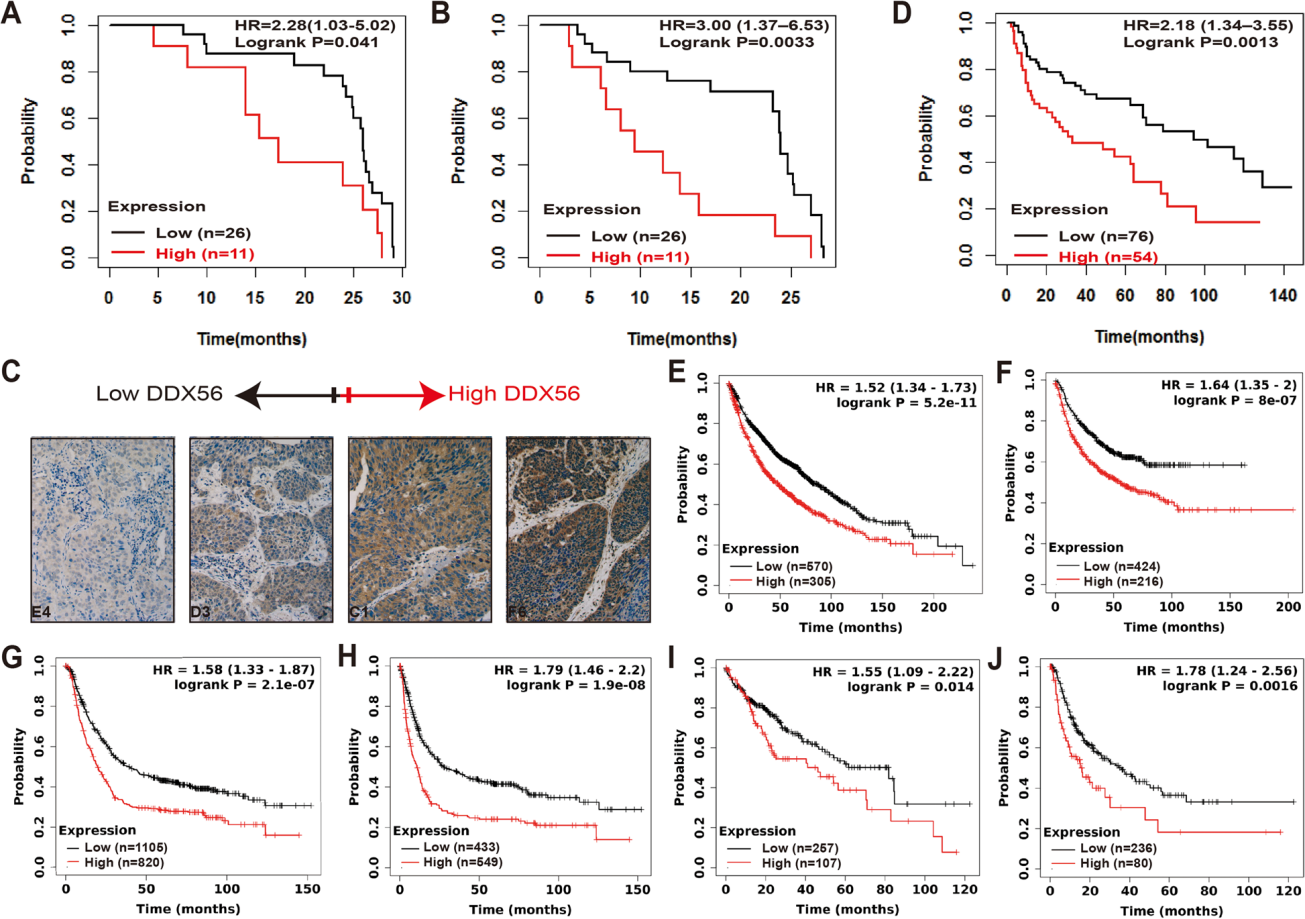
High DDX56 tumor expression is significantly associated with poor patient outcome in SqCLC and other cancers. **a**, **b** and **c** Immunohistochemical analysis showing that high DDX56 tumor protein level is associated with poor prognosis of SqCLC patients. Tissue microarrays (TMA) containing cores of 37 SqCLC were graded as exhibiting no, weak, moderate and strong DDX56 staining by a highly experienced pathologist blinded to all clinicopathologic data. Kaplan–Meier analysis comparing tumors graded as no or weak staining (low) versus those graded moderate and strong staining (high) showed that the overall survival (OS) (**a**) and recurrence-free survival (RFS) (**b**) of SqCLC patients with high DDX56 protein tumor expression is significantly lower than those with low DDX56 protein tumor expression. **c** Representative images of the different immunohistochemistry staining intensities. **d** Kaplan–Meier survival analysis of DDX56 in a published microarray gene expression dataset of 130 primary SqCLC tumor samples showed that high mRNA expression of DDX56 was significantly associated with poor OS of SqCLC patients. **e-j** Online survival analysis of the prognostic value of DDX56 mRNA expression in lung, gastric and liver cancers using KMplot (http://www.kmplot.com). DDX56 mRNA expression levels were shown to be significantly associated with OS (**e**) and progression-free survival (PFS) (**f**) in lung cancer patients (microarray data set, n = 1925). DDX56 mRNA expression levels were shown to be significantly associated with poor OS (**g**) and PFS (**h**) in gastric cancer patients (microarray data set, n = 875). DDX56 mRNA expression levels were shown to be significantly associated with OS (**i**) and PFS (**j**) in liver cancer patients (RNA-seq data set, n = 364)

To further study the correlation between DDX56 protein level and outcome in resected SqCLC patients, we collected a second independent cohort consisting of 56 SqCLC patients of whom 37 had complete follow-up data available. For this cohort, we analyzed the DDX56 level in primary tumors of the SqCLC patients by IHC. Tumors were divided into low (negative and weak IHC staining) or high (moderate and strong IHC staining) DDX56 protein expression groups. Log-rank test analysis showed that DDX56 protein levels significantly correlated with OS (HR = 2.28, 95% CI 1.03–5.02, *p* = 0.041; Fig. 2 a) and RFS (HR = 3.02, 95% CI 1.40–6.53, *p* = 0.0034; Fig. 2 b) in this validation cohort of SqCLC patients. Representative images of the different DDX56 IHC staining intensities are provided in Fig. 2 c and Additional file 1: Figure S1. Adjacent normal lung tissues from four of the patients exhibited no or weak staining (Additional file 1: Figure S2). Cox univariate and multivariate analysis of OS (Additional file 1: Table S6) and RFS (Table 1) in association with DDX56 protein levels and clinicopathologic characteristics, including age at surgery, stage, gender, tumor size, lymphovascular invasion status, tumor location and smoking history, showed that DDX56 was an independent prognostic factor of short RFS (HR = 2.74, 95% CI 1.07–7.02, *p* = 0.036) in patients with SqCLC.

**Table 1:** Cox univariate and multivariate analysis showing correlations between selected clinicopathologic characteristics and DDX56 IHC staining with recurrence-free survival in an independent SqCLC patient cohort (n = 56)

Variable	RFS Univariate Analysis HR (95% CI)	*p*	RFS Multivariate Analysis HR (95% CI)	*p*
Age (≥ 60/ < 60 years)	1.29 (0.64–2.58)	0.47	-	-
Sex (female / male)	1.36 (0.32–5.74)	0.68	-	-
Stage (IIIa / IIb / IIa / Ib/ Ia)	1.34 (1.01–1.77)	0.039	1.06 (0.76–1.48)	0.72
Tumor Size (> 3 cm)	1.44 (0.65–3.17)	0.36	-	-
Lymphovascular invasion (yes/no)	1.04 (0.54–2.01)	0.91	-	-
Tumor location (central/peripheral)	1.35 (0.66–2.76)	0.41	-	-
Smoke (yes/no)	0.95 (0.37–2.5)	0.92	-	-
DDX56 staining (strong or moderate/ weak or negative)	3.02 (1.40–6.53)	0.005	2.74 (1.07–7.02)	0.036

To further investigate the prognostic ability of DDX56 in SqCLC, we investigated the correlation between DDX56 gene expression and OS in a previously published gene microarray dataset of 130 SqCLC patients (34). The analysis revealed that higher DDX56 gene expression in primary SqCLC tumors significantly correlated with poor OS (HR = 2.18, 95% CI 1.34–3.55, *p* = 0.0017; Fig. 2 d). Multivariate analysis showed that SqCLC patients with high DDX56 tumor expression had a poor OS (HR = 2.06, 95% CI 1.26–3.37, *p* = 0.0040) regardless of their lymph node status (Additional file 1: Table S7).

To examine the correlation between DDX56 expression and outcome in lung cancer and other cancer types, we used the online survival analysis tool KMplotter [32, 33] and a dataset of 1925 lung cancer patients with available microarray data mRNA expression and clinical data. High DDX56 gene expression level was shown to be significantly associated with both OS and progression-free survival (PFS) in lung cancer (OS: HR = 1.52, 95% CI 1.34–1.73, *p* = 5.2e-11; Fig. 2 e; PFS: HR = 1.64, 95% CI 1.35–2.00, *p* = 8.0e-7; Fig. 2 f), Furthermore, multivariate analysis showed that DDX56 was an independent prognostic marker of OS and PFS in the lung cancer patients (HR = 1.57, 95% CI 1.32–1.87, *p* = 0.0071) and PFS (HR = 1.45, 95% CI 1.07—1.98, *p* = 0.017) after adjustment for grade, tumor size, lymph node status, gender and smoking history (Additional file 1: Table S8).

Analysis of the prognostic ability of DDX56 gene expression in gastric and liver cancer using datasets from 875 gastric (microarray data) and 364 liver (RNA-seq data) cancer patients with available mRNA expression and clinical data revealed that high DDX56 gene expression level was significantly associated with both OS and PFS in gastric cancer (OS: HR = 1.58, 95% CI 1.33–1.87, *p* = 2.1e-7; Fig. 2 g; PFS: HR = 1.79, 95% CI 1.46–2.20, *p* = 1.9e-8; Fig. 2 h) and liver cancer (OS: HR = 1.55, 95% CI 1.09–2.22, *p* = 0.014; Fig. 2 i; PFS: HR = 1.78, 95% CI 1.24–2.56, *p* = 0.0016; Fig. 2 j).

### DDX56 stimulates growth and migration of SqCLC cells in vitro and promotes xenograft tumor growth in vivo

**Fig. 3:**
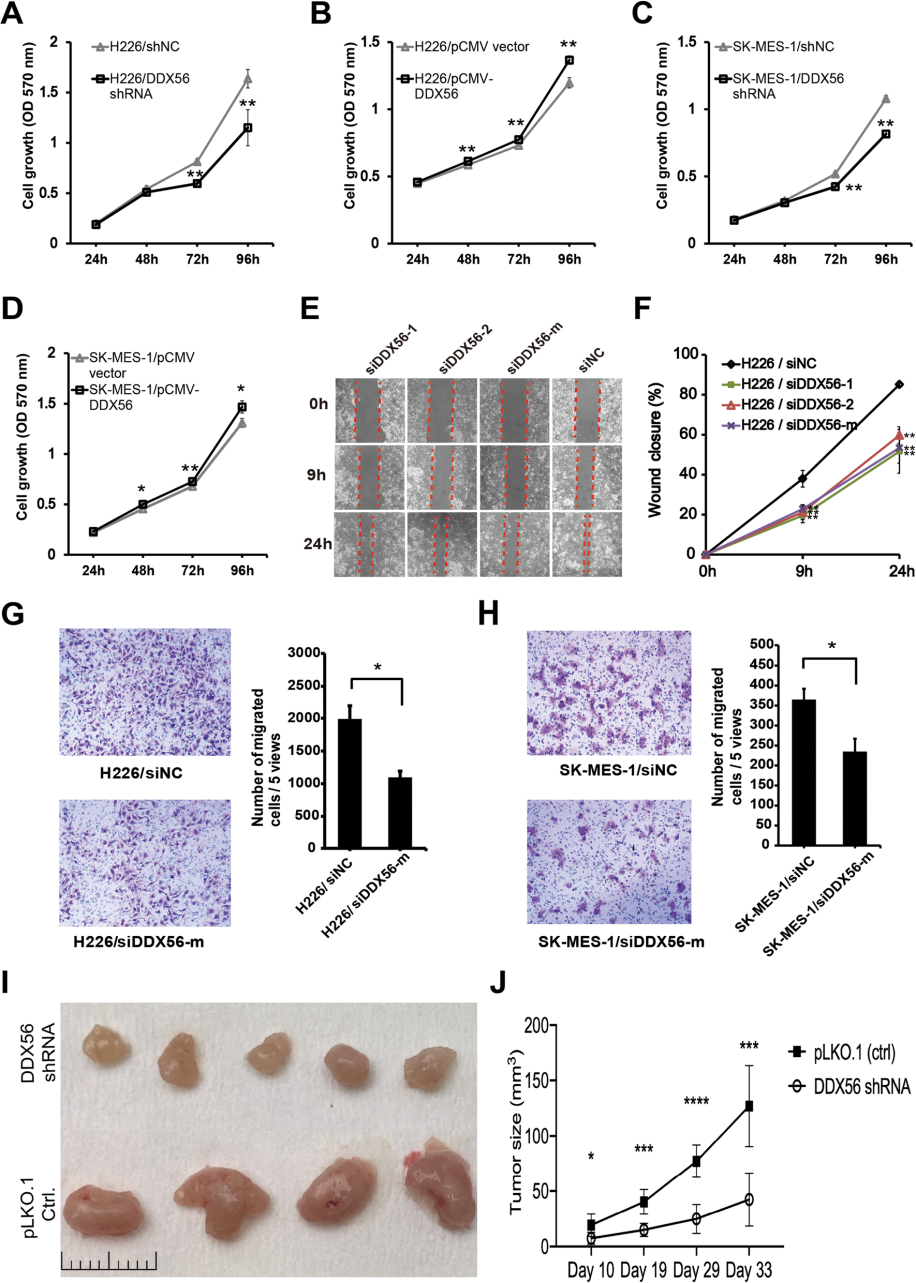
Reduction of DDX56 inhibits SqCLC cell growth, migration and tumor progression in a xenograft mouse model of SqCLC. **a** and **b** Analysis of growth of H226 (**a**) and SK-MES-1 (**b**) cells transfected with DDX56-specific or control shRNAs using a crystal violet assay. Cell growth of H226 and SK-MES-1 was significantly inhibited in DDX56 shRNA-transfected cells compared to control shRNA (shNC)-transfected cells (n = 4). **c** and **d** Analysis of growth of H226 and SK-MES-1 cells transfected with pCMV-DDX56 or control pCMV vectors using a crystal violet assay. Cell growth of H226 and SK-MES-1 was significantly enhanced in pCMV-DDX56-transfected cells compared to pCMV vector-transfected cells (n = 4). **e** and **f** Analysis of migration ability of H226 cells transfected with DDX56 or control siRNAs using wound healing assay. (**e**) Wound healing assay of H226 cells transfected with DDX56-specific or control siRNAs. (**f**) Quantification of wound closure by measuring the wound width of the % of the closure of original wound in triplicate plates. **g** and **h** Analysis of migration ability of H226 (**g**) and SK-MES-1 (**h**) cells transfected with DDX56-specific or control siRNAs using a transwell assay. Representative images of crystal violet-stained migrated H226 (200X) and SK-MES-1 (400X) cells on the membrane (left panel). Quantification of cell migration expressed by cell counting. Columns represent mean (n = 4). **i** and **j**, NOG CIEA were injected subcutaneously with H226 cells transfected with shDDX56 (n = 10) or shNC (n = 10) and monitored for H226 tumor growth at day 10, 19, 29 and 33 after injection (**i**). The tumor-bearing mice were sacrificed on day 33 and their tumors dissected. Representative tumors from DDX56 shRNA (n = 5) and control shRNA (n = 4) xenografts (**j**). Data are presented as mean ± SD. Two-sided Student’s t-test was used to compare the data. (*p < 0.05; **p < 0.01, ***p < 0.001, ****p < 0.0001). All data are representative of three independent experiments

To investigate the functional role of DDX56 in SqCLC cells, we performed knockdown of DDX56 in the human SqCLC cell lines H226 and SK-MES-1 and evaluated its effect on cell growth and migration. DDX56 silencing significantly reduced growth of H226 and SK-MES-1 cells at 48 and 72 h after transfection compared to control shRNA-transfected cells, as determined by crystal violet assay (Fig. 3 a and b; *p* < 0.01). Next, H226 and SK-MES-1 cells overexpressing DDX56 via transfection with pCMV-DDX56 or empty pCMV vector were tested in the same assay. Cells overexpressing DDX56 showed an increased growth rate compared to cells transfected with empty vector (Fig. 3 c and d). Overexpression and silencing of DDX56 were confirmed by Western blotting (Additional file 1: Figure S3).

The effect of DDX56 silencing on cell migration was assessed by wound healing assays and transwell migration. In both assays, siRNA-mediated DDX56 silencing significantly suppressed migration of SqCLC cells (Fig. 3 e–h). The potential effect of DDX56 silencing on SqCLC cell apoptosis was evaluated, but no significant alteration following 72-h culture in serum-free medium was observed (Additional file 1: Figure S4).

Since our in vitro data suggested an oncogenic role of DDX56 in SqCLC cells, we further elucidated its role in malignant tumorigenesis in vivo. Thus, we compared tumor growth of xenograft mouse models established from H226 cells stably transfected with either DDX56 shRNA (n = 10) or control shRNA (n = 10). As shown in Fig. 3 i, DDX56-silenced H226 xenograft tumors exhibited significantly decreased growth compared to control xenograft tumors. At endpoint, it was visually clear that the DDX56-depleted xenografts were smaller than the controls (Fig. 3 j). Taken together, our data indicate that DDX56 stimulates growth and migration of SqCLC cells in vitro and promotes tumor growth in vivo.

### DDX56 modulates the expression of Wnt signaling pathway-related genes

**Fig. 4:**
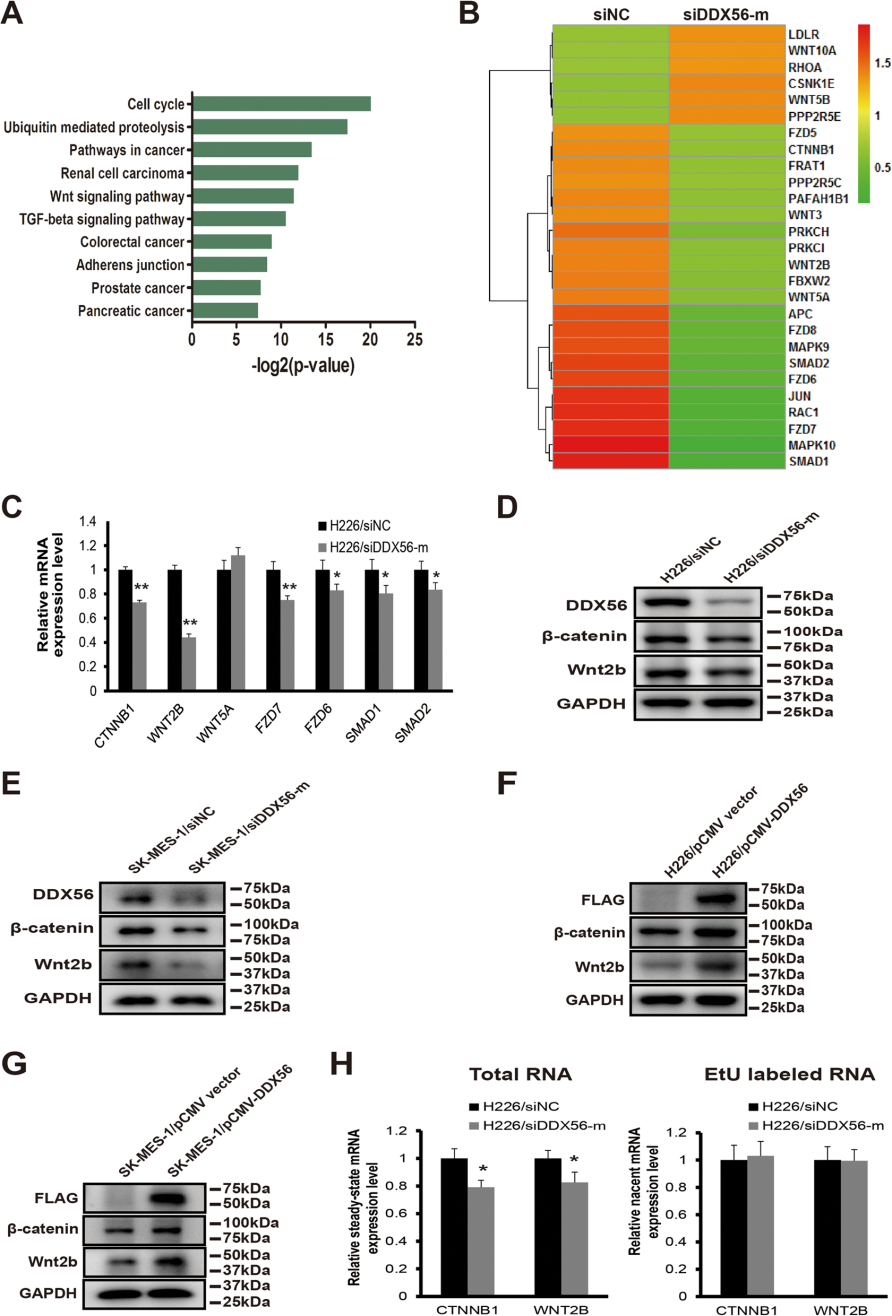
DDX56 post-transcriptionally regulates expression of the Wnt signaling pathway-related genes. **a** GO (KEGG) enrichment analysis of down-regulated mRNAs (> twofold) in a mRNA microarray experiment revealed that the KEGG terms “pathways in cancer” and “Wnt signaling pathway” were significantly enriched in down-regulated mRNAs in DDX56 siRNA- vs. control siRNA-transfected cells. **b** Heatmap showing WNT signaling-related genes differentially expressed (> twofold) between scramble siRNA (left) and DDX56 siRNA-treated (right) H226 cells in a mRNA microarray experiment. **c** qRT-PCR analysis of CTNNB1, WNT2B, WNT5A, FZD7, FZD6, SMAD1, and SMAD2 mRNA expression levels in DDX56-specific siRNA (siDDX56-mix) or negative control siRNA- (siNC) transfected H226 cells showed that DDX56 reduction significantly reduced mRNA expression of Wnt pathway-related genes CTNNB1, WNT2B, FZD7, FZD6, SMAD1, and SMAD2 except for WNT5A in H226 cells. **d** and **e** Western blot analysis of β-catenin, Wnt2b, and DDX56 protein expression level in H226 and SK-MES-1 cells transfected with DDX56-specific siRNAs or control siRNA, confirming reduced protein expression of β-catenin and Wnt2b following DDX56 reduction in H226 (**d**) and SK-MES-1 (**e**) cells. **f** and **g** Western blot analysis of the expression levels of β-catenin, Wnt2b and FLAG tag-fused DDX56 protein in H226 and SK-MES-1 cells transfected with pCMV-DDX56 or control pCMV vectors, confirming enhanced protein expression of β-catenin and Wnt2b following DDX56 overexpression in H226 (**f**) and SK-MES-1 (**g**) cells. GAPDH expression was used as a loading control. **h** Nascent transcript analysis showed no significant variation in the newly synthesized EtU-labeled RNA of CTNNB1 and WNT2B, whereas a significant decrease in the total (steady-state) RNA levels of CTNNB1 and WNT2B was observed after siRNA-mediated DDX56 knockdown in H226 cells. Gene expression levels were measured by qRT-PCR and standardized with GAPDH. Data are graphed as mean ± SD (n = 4). * *P* < 0.05

To identify putative targets of DDX56, we performed global gene expression profiling following siRNA-mediated DDX56 silencing in H226 cells and identified 2196 downregulated and 1478 upregulated transcripts (> twofold) in DDX56 siRNA-transfected cells compared to control siRNA-transfected cells. KEGG pathway enrichment analysis performed on both up- and down-regulated mRNAs (Additional file 1: Table S9) revealed that the KEGG terms “pathways in cancer” and “Wnt signaling pathway” were significantly enriched in downregulated mRNAs in DDX56 siRNA vs. control siRNA-transfected cells (Fig. 4 a and b). Since the Wnt signaling pathway has been shown to play a prominent role in maintaining cancer stemness as well as recurrence after surgical resection, we further investigated the role of DDX56 in the regulation of this pathway. Initially, we evaluated the influence of DDX56 silencing on mRNA levels of Wnt signaling pathway-associated genes, including CTNNB1, WNT2B, WNT5A, FZD7, FZD6, SMAD1 and SMAD2 in H226 cells. CTNNB1, WNT2B, FZD7, FZD6, SMAD1 and SMAD2 were significantly downregulated in DDX56 siRNA-transfected H226 as determined by qRT-PCR, while no change was observed for WNT5A (Fig. 4 c). Next, the β-catenin and Wnt2b protein levels in siRNA DDX56 siRNA- versus control siRNA-transfected H226 and SK-MES-1 cells were analyzed by Western blotting, showing that both proteins were reduced after DDX56 silencing (Fig. 4 d, e and Additional file 1: Figure S5A, B). Moreover, in agreement with above qRT-PCR data, the protein levels of β-catenin and Wnt2b were increased in pCMV-DDX56- versus pCMV vector-transfected H226 and SK-MES-1 cells (Fig. 4 f, g and Additional file 1: Figure S5C, D).

To further investigate whether the DDX56-regulated Wnt signaling pathway-related gene expression was regulated at the transcriptional or post-transcriptional level, we utilized the Click-iT® Nascent RNA Capture kit to label newly-transcribed RNA, which were subsequently isolated from the cells. Steady-state mRNA (total mRNA) of CTNNB1 and WNT2B was significantly decreased upon DDX56 silencing (Fig. 4 h), while no significant alteration in the EtU-labeled mRNAs was observed, indicating that CTNNB1 and WNT2B transcription is not affected by DDX56 silencing (Fig. 4 i). Thus, taken together, our findings suggest that DDX56 post-transcriptionally upregulates multiple genes in the Wnt signaling pathway.

### miR-378a-3p is a mediator in the post-transcriptional regulation of Wnt pathway-related genes by DDX56

**Fig. 5:**
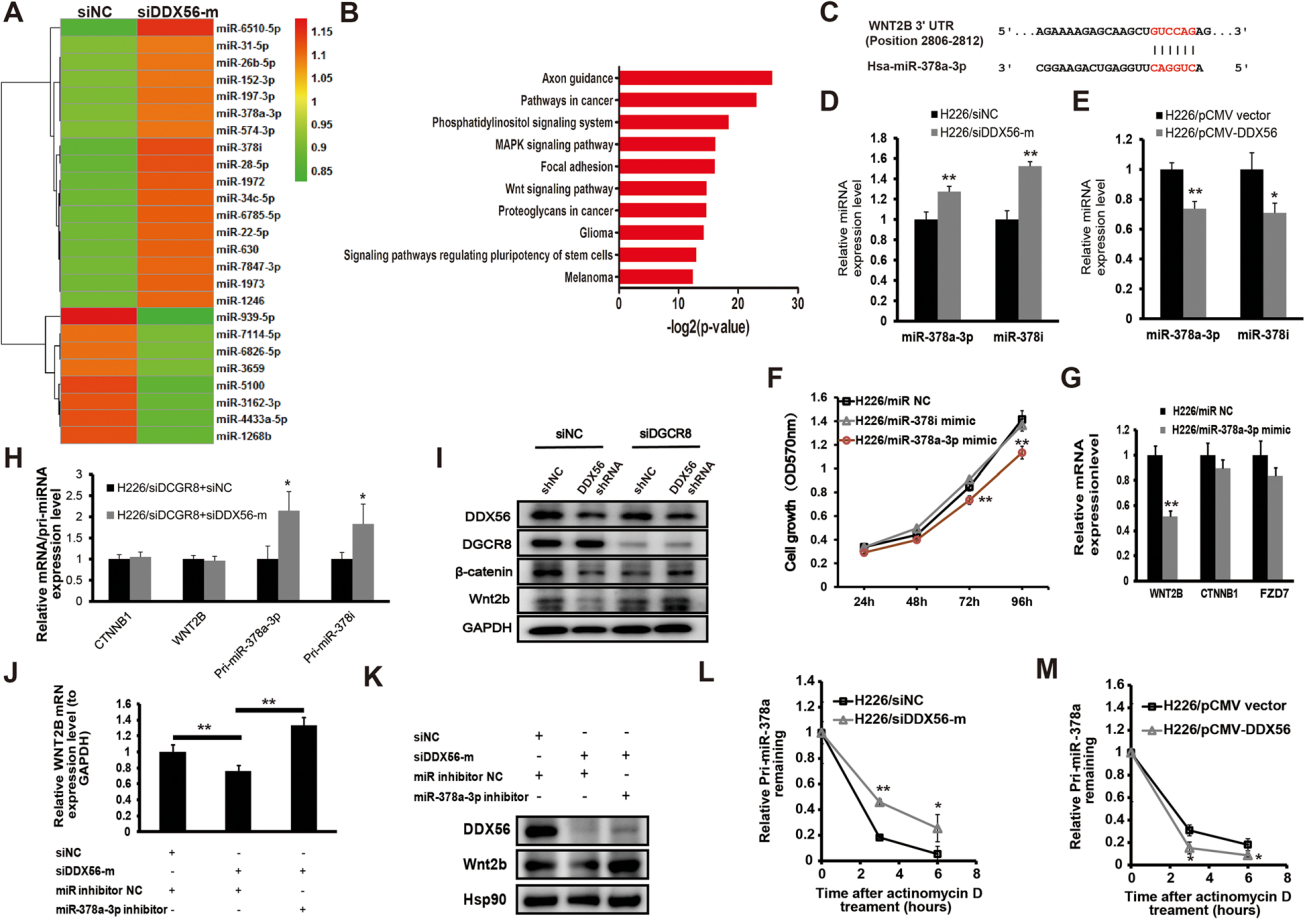
miR-378a-3p as a downstream effector of DDX56 involved in regulation of WNT2B expression in SqCLC cells. **a** Heatmap showing differentially-expressed miRNAs (≥ 1.2-fold) between scramble siRNA (left) and DDX56 siRNA-treated (right) H226 cells in a miRNA microarray experiment. **b** GO (KEGG) enrichment analysis of predicted targets of upregulated miRNAs (≥ 1.2-fold) in a miRNA microarray experiment revealed that the KEGG terms “pathways in cancer” and “Wnt signaling pathway” were also significantly enriched in predicted targets of up-regulated miRNAs in DDX56 siRNA- vs. control siRNA-transfected cells. **c** Predicted binding site of human miR-378a-3p to the 3’UTR of WNT2B by TargetScan. **d** and **e** qRT-PCR analysis of mature miRNA levels of miR-378a-3p and miR-378i in DDX56 silenced or overexpressed H226 cells compared to control cells. **d** H226 cells were transfected with DDX56-specific siRNA or control siRNA. **e** H226 cells were transfected with pCMV-DDX56 vector or pCMV empty vector. U6B was used for normalization of the mature miRNA expression data. (n = 3). **f** Analysis of growth of H226 cells transfected with miR-378a-3p mimic, miR-378i mimic or negative control miRNA mimic as determined by a crystal violet assay. (n = 4). **g** qRT-PCR analysis of WNT2B mRNA expression level in H226 cells transfected with miR-378a-3p mimic or control miRNA. GAPDH was used for normalization. (n = 3). **h** qRT-PCR analysis of mRNA expression of WNT2B and CTNNB1 and primary miRNA expression of miR-378a-3p and miR-378i in H226 cells co-transfected with DGCR8 siRNA and control siRNA or DGCR8 siRNA and DDX56 siRNA. GAPDH was used for normalization. **i** Representative Western blot analysis of Wnt2b and β-catenin protein expression levels in H226 cells co-transfected with control siRNA and control shRNA or control siRNA and DDX56 shRNA or DGCR8 siRNA and control shRNA or DGCR8 siRNA and DDX56 shRNA. GAPDH was used as a loading control. (n = 3). **j** qRT-PCR analysis of WNT2B mRNA expression level in H226 cells co-transfected with control miRNA and control siRNA or control miRNA and DDX56 siRNA or miR-378a-3p mimic and DDX56 siRNA. GAPDH was used for normalization. **k** Representative Western blot analysis of Wnt2b protein expression level in H226 cells co-transfected with control miRNA and control siRNA or control miRNA and DDX56 siRNA or miR-378a-3p mimic and DDX56 siRNA. Expression of a housekeeping gene, Hsp90 was used as a loading control. (n = 3). **l** and **m** Pri-miRNA stability assay carried out using H226 cells. **l** H226 cells were transfected with DDX56-specific siRNA (si-DDX56-mix) or control siRNA for 48 h, then treated with the transcription inhibitor actinomycin D. Primary miR378a were quantitated by qRT-PCR normalized to 18 s rRNA at 0, 3, and 6 h. (n = 3). **m** H226 cells were transfected with pCMV-DDX56 or empty pCMV vector for 48 h, then treated with the transcription inhibitor actinomycin D. Primary miR378a were quantitated by qRT-PCR, normalized to 18 s rRNA at 0, 3, and 6 h. (n = 3). Primary miR-378a expression level at 0 h was defined as 1. Each experiment was performed independently at least twice and results are presented as mean ± SD. Two-sided Student’s t-test was used to analyze the data. (*p < 0.05; **p < 0.01)

DEAD-box RNA helicases are known to participate in miRNA biogenesis. To examine whether miRNAs were involved in the post-transcriptional regulation of Wnt signaling by DDX56, we performed miRNA expression profiling following siRNA-mediated DDX56 silencing in H226 cells. Our miRNA microarray assessment identified 5 downregulated and 17 upregulated miRNAs (≥ 1.2-fold) in DDX56 siRNA-transfected H226 cells compared to control siRNA-transfected cells (Fig. 5 a, Additional file 1: Table S10). KEGG pathway enrichment analysis was performed on predicted targets of both up- and down-regulated miRNAs identified in the microarray assays (Additional file 1: Table S11). Interestingly, the KEGG terms “pathways in cancer” and “Wnt signaling pathway” were significantly enriched in predicted targets of upregulated miRNAs in DDX56 siRNA vs. control siRNA-transfected cells (Fig. 5 b), which implied that DDX56 may promote expression of Wnt signaling-related genes by repressing the subset of miRNAs.

Two miR-378 family miRNAs, miR-378i and miR378a-3p, were upregulated in DDX56 siRNA- vs control siRNA-transfected H226 cells. miR-378 family members have been shown to inhibit cell growth and inactivate the Wnt/β-catenin pathway [34, 35], and in silico target prediction analysis by TargetScan revealed that the WNT2B transcript was predicted to contain a miR-378a-3p target site on 3' UTR (Fig. 5 c). Thus, we evaluated whether the two miR-378 family miRNAs acted as mediators for the regulation of Wnt signaling pathway by DDX56. Initially, to confirm the results obtained from the miRNA microarray and further investigate the expression levels of their primary transcripts, we used qRT-PCR to examine the expression of primary and mature miR-378i and miR378a-3p miRNAs in DDX56 silenced or overexpressed H226 cells compared to control cells. As shown in Fig. 5 d and e, miR-378i and miR378a-3p were upregulated in DDX56 siRNA vs control siRNA-transfected cells, but downregulated in pCMV-DDX56 vector vs pCMV empty vector transfected cells. In accordance with the expression of the mature miRNAs, the primary miR-378i and miR378a-3p were also upregulated in DDX56 siRNA vs control siRNA-transfected cells (Additional file 1: Figure S6). Subsequently, the role of the two miRNAs on cancer cell growth were evaluated and showed that the miR-378a-p3 mimic, but not the miR-378i mimic, significantly reduced cell growth in H226 cells compared to mock-transfected cells (Fig. 5 f). Examining mRNA expression of CTNNB1, WNT2B and FZD7 in H226 cells 48 h after transfection of the miR-378a-3p mimic or miRNA negative control by qRT-PCR revealed that WNT2B was significantly reduced in miR-378a-3p mimic-transfected cells compared to control cells, while no change in CTNNB1 and FZD7 levels were observed (Fig. 5 g).

To verify the hypothesis that miRNAs are involved in the post-transcriptional regulation of genes/proteins in the Wnt signaling pathway by DDX56, we performed co-knockdown of DDX56 and DGCR8 in H226 cells. DGCR8 is a key component of the microprocessor complex and essential for processing of pri-miRNAs both in vitro and in vivo. Co-knockdown of DGCR8 in DDX56 siRNA-transfected and control siRNA-transfected cells was sufficient to abolish the DDX56-mediated regulation of CTNNB1 and WNT2B mRNAs, but not the regulation in the primary miRNAs (Fig. 5 h). This observation was confirmed by Western blot analysis of β-catenin and Wnt2b in both DDX56 stably silenced and control H226 cells co-transfected with DGCR8 or control siRNAs (Fig. 5 i).

To further validate the role of miR-378a-3p as a mediator in the regulation of WNT2B by DDX56, we co-transfected H226 cells with either 1) miR-378a-3p inhibitor and DDX56 siRNA, 2) miRNA inhibitor negative control and DDX56 siRNA or 3) miRNA inhibitor negative control and siRNA negative control, and evaluated mRNA and protein expression of WNT2B by qRT-PCR and Western blotting. As shown in Fig. 5 j and k, the reduced expression of WNT2B caused by transfection of DDX56 siRNA in H226 cells was significantly reversed both at the mRNA and protein levels by co-transfection with the miR-378a-3p inhibitor, strongly suggesting that miR-378a-3p is a downstream mediator negatively regulated by DDX56, leading to decreased expression of WNT2B.

**Fig. 6:**
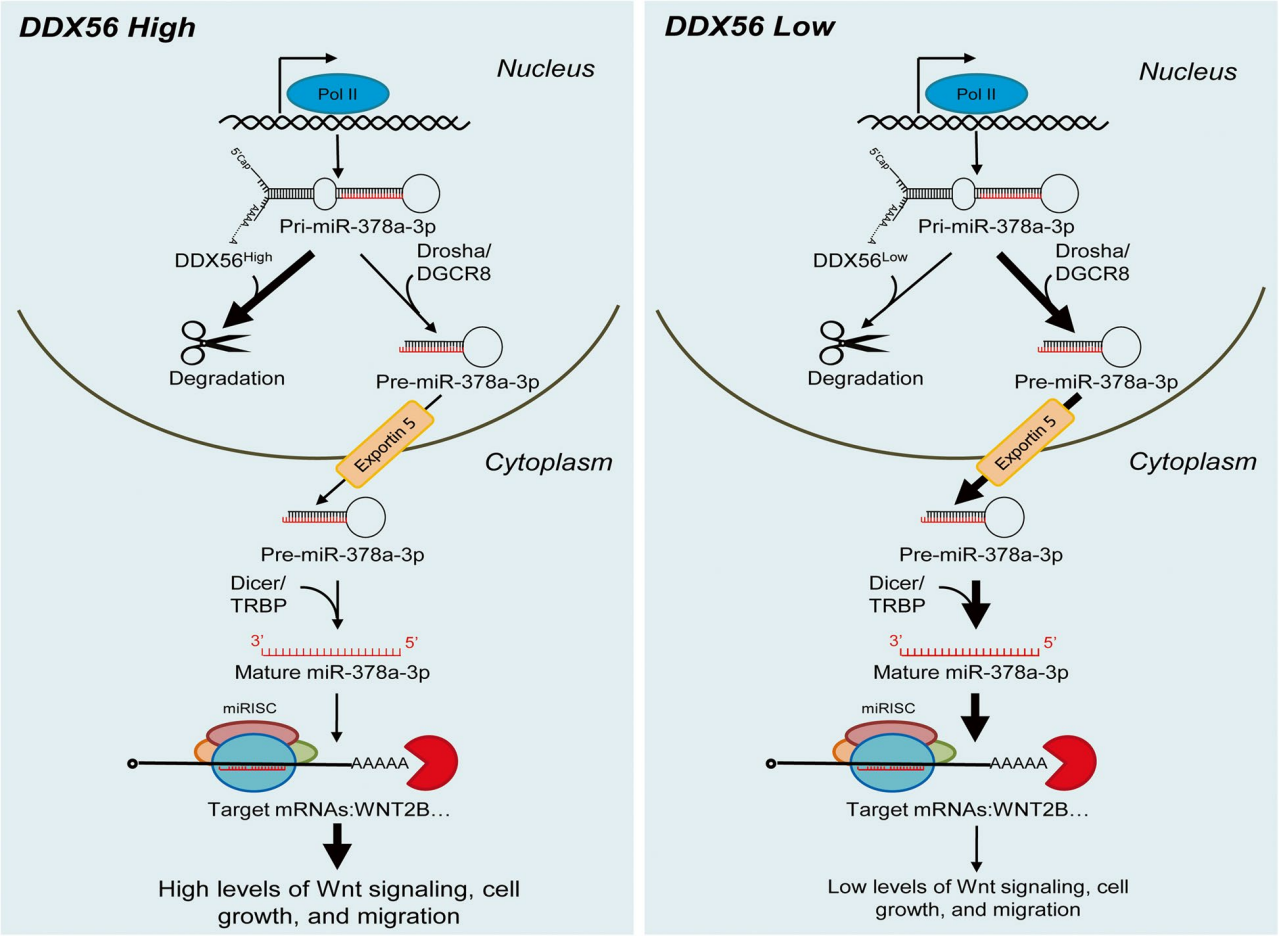
A proposed model for DDX56 regulation of miR-378a-3p and subsequent modulation of WNT signaling in SqCLC cells. A proposed working model showing the DDX56/miR-378a-3p/Wnt2b signaling axis and its roles in SqCLC cells with high (left) or low (right) DDX56 expression. Thick arrows indicate more flow; thin arrows indicate less flow

Since members of the DEAD-box protein family have been shown to participate in primary miRNA processing and mRNA decay, we investigated the potential effect of DDX56 on regulation of primary miR-378a by a RNA stability assay using H226 cells transfected with DDX56 siRNA, siRNA negative control, pCMV-DDX56 vector and pCMV empty vector. In this assay, the cells were treated 48 h after transfection with the transcription inhibitor actinomycin D, and primary miR-378a levels were quantitated by qRT-PCR after 0, 3 and 6 h. As shown in Fig. 5 l, the degradation of primary miR-378a in DDX56 siRNA-transfected cells was significantly lower than in control siRNA-transfected cells at 3 h. In agreement, the degradation of primary miR-378a in pCMV-DDX56 vector-transfected cells was significantly greater than in pCMV empty vector-transfected cells at 6 h (Fig. 5 m). Combined, these results suggest that DDX56 facilitates degradation of primary miR-378a, leading to down-regulation of mature miR-378a-3p, thereby derepressing expression of the WNT2B gene (Fig. 6).

## Discussion

Local and distant recurrences are the primary causes of poor survival in SqCLC patients even after successful resection [36, 37]. Defining the molecular mechanism leading to early recurrence following primary resection and identification of protein markers for risk prediction are essential to guide personalized treatment decisions. In this study, we identified 131 differentially-expressed proteins in SqCLC tumors of patients exhibiting ER vs. LR using mass spectrometry-based proteomics, including DDX56. Importantly, we show that the high DDX56 expression level correlates with poor prognosis of patients with SqCLC and other cancer types and is an independent prognostic marker of RFS in SqCLC. Analysis of the functional role of DDX56 in cancer cells revealed that DDX56 modulates expression of multiple Wnt signaling-related genes, promotes growth and migration of SqCLC cells in vitro and xenograft tumor growth in vivo. Together, these findings suggest an oncogenic role of DDX56, which may contribute to the postoperative early recurrence of SqCLC.

Our finding that DDX56 regulates Wnt signaling is intriguing, as aberrant Wnt signaling is frequently observed in cancer and linked to cancer recurrence. Patient-specific pathway analysis of primary tumor of SqCLC using mRNA expression data from The Cancer Genome Atlas (TCGA) identified the Wnt signaling pathway as one of the most significant [38], and it has been shown that Wnt pathway activation significantly increases the risk of recurrence after resection of stage I NSCLC [39]. However, few studies have assessed the upstream regulators of the aberrant Wnt signaling in SqCLC recurrence. Our data showed that the pivotal genes in Wnt signaling pathway, including CTNNB1 and WNT2B, encoding β-catenin and Wntb2 were post-transcriptionally regulated by DDX56. Interestingly, a recent study on prostate cancer showed that β-catenin is crucial for hTERT-mediated CSC traits [40]. Another study demonstrated increased levels of Wnt ligands in lung adenocarcinomas compared to adenomas and normal lung tissue, and proposed that a subset of cancer cells gave rise to a Wnt-producing niche, essential for the maintenance of Wnt responder stem-like cells and driving progression in lung adenocarcinoma [41]. Thus, we inferred that increased DDX56 contributes to the early recurrence of SqCLC, perhaps via regulation of the Wnt signaling pathway, thereby maintaining CSC traits.

Pathway analysis of predicted targets of upregulated miRNAs following DDX56 silencing suggested that miRNAs may be involved in DDX56 regulation of the Wnt signaling pathway. The co-knockdown experiments confirmed that the DDX56-mediated regulation on β-catenin and Wnt2b is dependent on presence of DGCR8, which is essential for maturation of miRNAs. miR-378 family members, especially miR-378a-3p, have been proposed to function as tumor suppressors [42] by suppressing Wnt/β-catenin signaling activation via targeting WNT10a and DVL2 [43, 44]. These results are in accordance with our findings that a miR-378a-3p mimic inhibits cell growth and repress WNT2B mRNA expression. However, it is noteworthy that the gene expression of CTNNB1 and FZD7 was slightly, but not significantly, repressed by miR-378a-3p mimic in vitro. As shown by miRNA microarray analysis, DDX56 downregulates the expression of a subset of miRNAs targeting Wnt signaling. Thus, whether other miRNAs or factors are involved in the DDX56-mediated regulation of CTNNB1 and FZD7 needs further study. Our study showed that DDX56 silencing upregulated primary miR-378a expression, and that DDX56 promoted primary miR-378a degradation. A more recent study showed that DDX56 can induce intron retention of WEE1, a cell cycle-related gene, in colorectal cancer cell [27]. Intron-retaining pre-mRNA transcripts are susceptible to degradation by nonsense-mediated mRNA decay (NMD) [45]. Meanwhile, GO analysis of interaction protein data from a previous study by Ewing et al. revealed that the GO term “nuclear-transcribed mRNA catabolic process, nonsense-mediated decay” was significantly enriched (Benjamini–Hochberg adjusted *P* value = 1.5E-22) among the DDX56-interacting proteins (data not shown) [46]. Thus, we inferred that DDX56 affects miR-378a-3p expression through promoting primary miRNA degradation, probably via the NMD pathway, although the molecular mechanism underlying this regulation requires further investigation.

Our study showed that the differential expression of DDX56 between ER and matched LR patients was only observed in tumor tissues and not in adjacent normal tissues that exhibited no or weak DDX56 staining by IHC. This implies that the increased expression of DDX56 is under tumor-specific regulation. Recently, several compounds that inhibit DDX3 by binding to the DDX3 ATP binding domain or RNA binding site have been investigated for their anti-cancer properties [47], which gives hope that DDX56 may also be targetable with small molecules and used in the treatment of SqCLC.

## Conclusion

In summary, we identified the differentially-expressed proteins associated with time to recurrence of resected SqCLC using quantitative proteomics. Among the identified candidate proteins, we found that high DDX56 expression in primary tumors correlated with poor OS and RFS of SqCLC patients. Functional studies revealed that DDX56 plays an oncogenic role in SqCLC, likely through miRNA-mediated post-transcriptional regulation of the Wnt signaling pathway. Our data show that DDX56 is an independent prognostic marker that can identify patients at high risk of postoperative SqCLC early recurrence who may benefit from additional postoperative treatment. As Wnt signaling is active in DDX56 high SqCLC tumors, Wnt signaling-targeted therapies may be particularly useful in these patients.

## Supplementary Information

**Additional file 1.**

**Additional file 2.**

**Additional file 3.**

## Data Availability

All data generated during this study are included in this published article and its supplementary files. Mass spectrometry proteomics data have been deposited to the ProteomeXchange Consortium via the PRIDE partner repository with the dataset identifier PXD009383. mRNA microarray and miRNA microarray data have been deposited in the ArrayExpress database at EMBL-EBI under accession numbers E-MTAB-6675 and E-MTAB-6676, respectively.
