# Supplementary material for: DDX56 modulates post-transcriptional Wnt signaling through miRNAs and is associated with early recurrence in squamous cell lung carcinoma
Source: Mol Cancer. 2021 Aug 26;20:108. doi: 10.1186/s12943-021-01403-w (PMC8393456; doi:10.1186/s12943-021-01403-w)
Supplement: Supplementary file 1 — Additional file 1. [file 12943_2021_1403_MOESM1_ESM.pdf]

DDX56 modulates post-transcriptional Wnt signaling through miRNAs and is associated with early recurrence in squamous cell lung carcinoma

Qingqing Wu, Xiaoyang Luo, Mikkel G Terp, Qingrun Li, Yuan Li, Lei Shen, Ying Chen, Kirstine Jacobsen, Trever G. Bivona, Haiquan Chen, Rong Zeng, Henrik J. Ditzel.

Supplementary Methods

Proteomic analysis

Tumor tissue samples from paired SqCLC patients in cohort1 were homogenized in SDT lysis buffer (4% SDS, 0.1M Tris-HCl pH 7.6, 0.1M DTT) using a Tissue Lyser II (Qiagen). Protein concentrations were determined by tryptophan fluorescence emission at 350 nm using an excitation wavelength of 295 nm [1]. A modified Filter Aided Sample Preparation (FASP) (compatible with iTRAQ labeling) was used for tissue digestion [2]. iTRAQ (isobaric tagging for relative and absolute quantification)-based quantitative proteomics was performed to determine the differential expression of proteins between the two patient tumor groups. To reduce technical bias of different channels, each two pairs of samples were labeled with 4-plex iTRAQ reagents (Applied Biosystems) in both orders (forward and reverse). The iTRAQ-labeled samples were mixed and fractionated on Agilent 1100 HPLC using SCX column. Six fractions were eluted using pH gradient elution buffer and desalted. The SCX-fractionated peptides were re-suspended in 0.1% FA solution and separated on a self-packed reversed phase tip column (75 μm × 150 mm; C18, 3 μm) using a 240 min gradient at a flow rate of 300 nL/min. LTQ Orbitrap velos was conducted in data-dependent acquisition mode

using a “high–high” strategy (high mass accuracy and resolution both on precursor and product ion mass). Full MS scans and HCD (higher energy collisional dissociation)-MS/MS scans for the ten most intense ions in each full MS scan were acquired at a resolution of 30,000 and 7500, respectively.

Protein identification was performed using Mascot 2.2.2 (Matrix Science) software. Each MS/MS spectrum searched against a concatenated version of the forward and reversed human UniProt fasta database. Maximum false discovery rates (FDR) were 0.01 at both peptide and protein levels. Quantitative ratios for peptides and proteins were obtained using Buildsummary software [3]. For the protein ratio, the median was calculated over all distinct peptides assigned to the protein subgroup, and both forward and reverse order quantitative data were considered. A median protein iTRAQ ratio of each replicates was normalized to 1.

The entire statistical analysis for the proteomic samples was based on the log2-values of the protein ratios. The Wilcoxon signed rank test was performed using R (version 3.2.0) to identify significant differentially-expressed proteins between the two patient groups, and the p-value was calculated. Both fold-change and p-values were considered in data analysis.

Cell culture and transfection

The human SqCLC cell line H226 and SK-MES-1 were purchased from ATCC and the Cell Bank of Type Culture Collection of Chinese Academy of Sciences (CBTCCCAS), respectively. H226 cells were grown in RPMI 1640 medium (61870036, Invitrogen)

with 10% FBS, and SK-MES-1 cells were grown in MEM medium (11090081, Invitrogen) with 10% FBS, 1% Glutamax (35050061, Invitrogen), 1% Non-essential Amino Acids (11140050, Invitrogen), and 1% Sodium Pyruvate 100 mM Solution (11360070, Invitrogen). All cells were maintained at 37 °C in humidified air with 5% CO2. All cell lines were free of mycoplasma contamination at the time of the experiments as determined by MycoAlert™ Mycoplasma Detection Kit. DDX56 siRNA #1 (SI00361781), DDX56 siRNA #2 (SI04238157), and negative control siRNA (SI03650318) were purchased from Qiagen (Hilden, Germany). DGCR8 siRNA and negative control siRNA were purchased from Genepharma (Shanghai, China). Transient knockdown of DDX56 was performed using two different DDX56 siRNAs (siDDX56-1 and siDDX56-2) individually or in combination (siDDX56-m) and compared to cells transfected with a scrambled siRNA (negative control, siNC). Stable knockdown of DDX56 in H226 or SK-MES-1 cells was performed by using MISSION shRNA lentiviral transduction particles targeting DDX56 (SHCLNV-NM_019082, Sigma-Aldrich) according to the manufacturer's instruction. MISSION pLKO.1-puro non-target shRNA control transduction particles (SHC016V, Sigma-Aldrich) were used to generate a control H226 or SK-MES-1 cell line. miR-378i mimic, miR-378a-3p mimic, miRNA mimic negative control, miRNA-378a-3p inhibitor and miRNA inhibitor negative control were purchased from Genepharma.

Western blotting

Tissues were homogenized in SDT lysis buffer (4%SDS, 0.1M Tris-HCl pH7.6, 0.1 M

DTT) using a Tissue Lyser II (Qiagen). Protein concentrations were determined by tryptophan fluorescence emission at 350 nm using an excitation wavelength of 295 nm. Cells were harvested using a cell scraper and lysed in RIPA lysis buffer (50 mM Tris 7.5, 150 mM NaCl, 1% NP-40, 0.5% sodium deoxycholate, 0.1%SDS). Total protein concentration was quantified by BCA assay. The extracted proteins from tissue or cell samples were separated on 12% SDS-PAGE and electroblotted onto PVDF membranes, which were then blocked in TBST buffer (Tris-buffered saline containing 0.1% Tween-20) containing 5% BSA for 1 hour at room temperature, and incubated with primary antibody anti-DDX56 (1:1000, sc-393078, Santa Cruz, CA, USA), anti-β-actin (1:5000, ab6276, Abcam), anti-GAPDH(1:3000, sc-25778, Santa Cruz, CA, USA), anti-HSP90 (1:1000, #4874, Cell Signaling Technology), anti-β-Catenin (1:1000, #8480, Cell Signaling Technology), or anti-Wnt-2b (1:200, sc-166502, Santa Cruz, CA, USA) in TBST buffer containing 5% BSA overnight at 4°C. The membranes were then washed with TBST and incubation with goat anti-mouse, goat anti-rabbit, or chicken anti-goat HRP-conjugated secondary antibody (Santa Cruz) in TBST buffer for 2 hours at room temperature, followed by washing in TBST buffer prior to visualization of immunoreactive bands using an ECL Prime Western Blot kit (GE Healthcare) and ImageQuant LAS4000(Fujifilm).

Tissue microarray (TMA) and immunohistochemistry (IHC)

TMA sections were deparaffinized, rehydrated, and endogenous peroxidase quenched with 0.3% hydrogen peroxide (v/v). Antigen retrieval was performed by heating the

sections for 10 min in antigen retrieval reagents (Beyotime Institute Biotech, Shanghai, China) in a microwave oven. After antigen retrieval, sections were blocked in 4% horse serum for 1 hour at room temperature, and incubated with anti-DDX56 (1:200, sc-393078, Santa Cruz, CA, USA) overnight at 4°C. After a rinse with PBST solution, the sections were incubated sequentially with HRP-rabbit anti-mouse antibody and DAB reagent (Dako Real Envision, K5007, CA,USA). Finally, sections were counterstained with Hematoxylin QS (Vector Labs, H3404, CA, USA).

Cell growth assay

Cells transfected with DDX56-specific siRNAs, control siRNAs, pCMV-DDX56 or empty pCMV vector were seeded into 24-well plates (4x10^4 per well) and, 24, 48 and 72 hours after transfection, cells were stained with 0.5% crystal violet stain solution. For miRNA mimic experiments, cells transfected with miR-378a-3p, miR-378i and negative control mimic (4x10^4 per well) were seeded into 24-well plates, stained with 0.5% crystal violet stain solution 24, 48, 72 and 96 hours after transfection, and subsequently washed with distilled water and dried in a fume hood. The stained dry cells in each well were dissolved in citrate buffer (0.1M sodium citrate in 50% ethanol) and absorbance values at 570 nm were measured using a Sunrise microplate Reader (Tecan Trading AG, Switzerland) or SpectraMax i3 Multi-Mode Microplate Reader (Molecular Devices, Sunnyvale, CA).

Transwell migration assay

Cell migration was assessed using Transwell chambers (8.0-um pore size; Corning, New York, NY). H226 and SK-MES-1 cells were transfected with DDX56-specific siRNAs or control siRNA, after which cell migration was assessed using Transwell chambers (8.0-um pore size; Corning, New York, NY). Each assay condition was performed in triplicate and performed 3 separate times. The migrating cells were counted and photographed in 5 random fields within an insert under a phase contrast microscope.

Wound healing assay

Cell migration was assessed by the ability of the cells to migrate into a cell-free area. Briefly, H226 cells transfected with DDX56-specific siRNAs or control siRNA ($2x10^5$ per well) were seeded into 12-well plates in triplicate. Cells were grown for about 24 hours to reach ~90% confluence and the monolayer was scratched with a sterile 200μl pipet tip across the center of the well. After wash, the wells were replenished with fresh medium and grown in an incubator. Microscope images were obtained at 0, 3, 6, 9 and 24 hours after scratch. The gap distance was measured from 3 scratches for each condition. The gap distance was measured from 3 scratches for each condition.

Apoptosis assay

A cell death detection ELISA kit (Roche) was used to investigate the effects of DDX56 reduction in H226 cells on serum deprivation-induced apoptosis. H226 cells transfected

with DDX56-specific siRNAs or control siRNA were seeded in 24–well plates (1x105 cells per well) in triplicate. After overnight incubation, the cells were deprived of serum for 72 hours and cell apoptosis was measured in accordance with the manufacturer's instructions.

Capture of nascent RNAs

The Click-it Nascent RNA Capture Kit (Invitrogen) was used to capture the nascent RNAs. Briefly, H226 cells were incubated with 0.5 mM 5-ethynyl-uridine (EtU) for 30 min, and EtU was incorporated into newly synthesized RNA. Total RNA was prepared with TRIzol reagent (Invitrogen). The EtU-labeled RNAs were biotinylated and captured by using the kit according to the manufacturer's instructions. The captured EtU-labeled and total RNA was subjected to analysis by qRT–PCR.

mRNA microarray

H226 cells transfected with DDX56 siRNA and control siRNA were incubated for 48 hours and total RNA isolated and used for the microarray analysis using Affymetrix Human U133 Plus 2.0 Arrays. Slides were scanned by GeneChip® Scanner 3000 (Cat#00-00212, Affymetrix, Santa Clara, CA, US) and Command Console Software 4.0 (Affymetrix, Santa Clara, CA, US) with default settings. Raw data were normalized by MAS 5.0 algorithm, Affy packages in R. Differentially-expressed mRNAs were identified by arbitrarily setting the threshold at a fold change of 2.

miRNA microarray

H226 cells transfected with DDX56 siRNA and control siRNA were incubated for 48 hours and miRNA isolated using miRNeasy mini kit (Qiagen) according to the manufacturer's instructions. miRNA expression profiling was conducted using the Agilent Human miRNA (8×60K) Microarray (Agilent Technologies). The arrays were washed and scanned with a laser confocal scanner (G2565BA, Agilent Technologies, Palo Alto, CA) according to the manufacturer's instructions. The intensities of fluorescence were calculated by Feature extraction software (Agilent Technologies). Differentially-expressed miRNAs were identified by arbitrarily setting the threshold at a fold change of 1.2.

Supplementary Figures

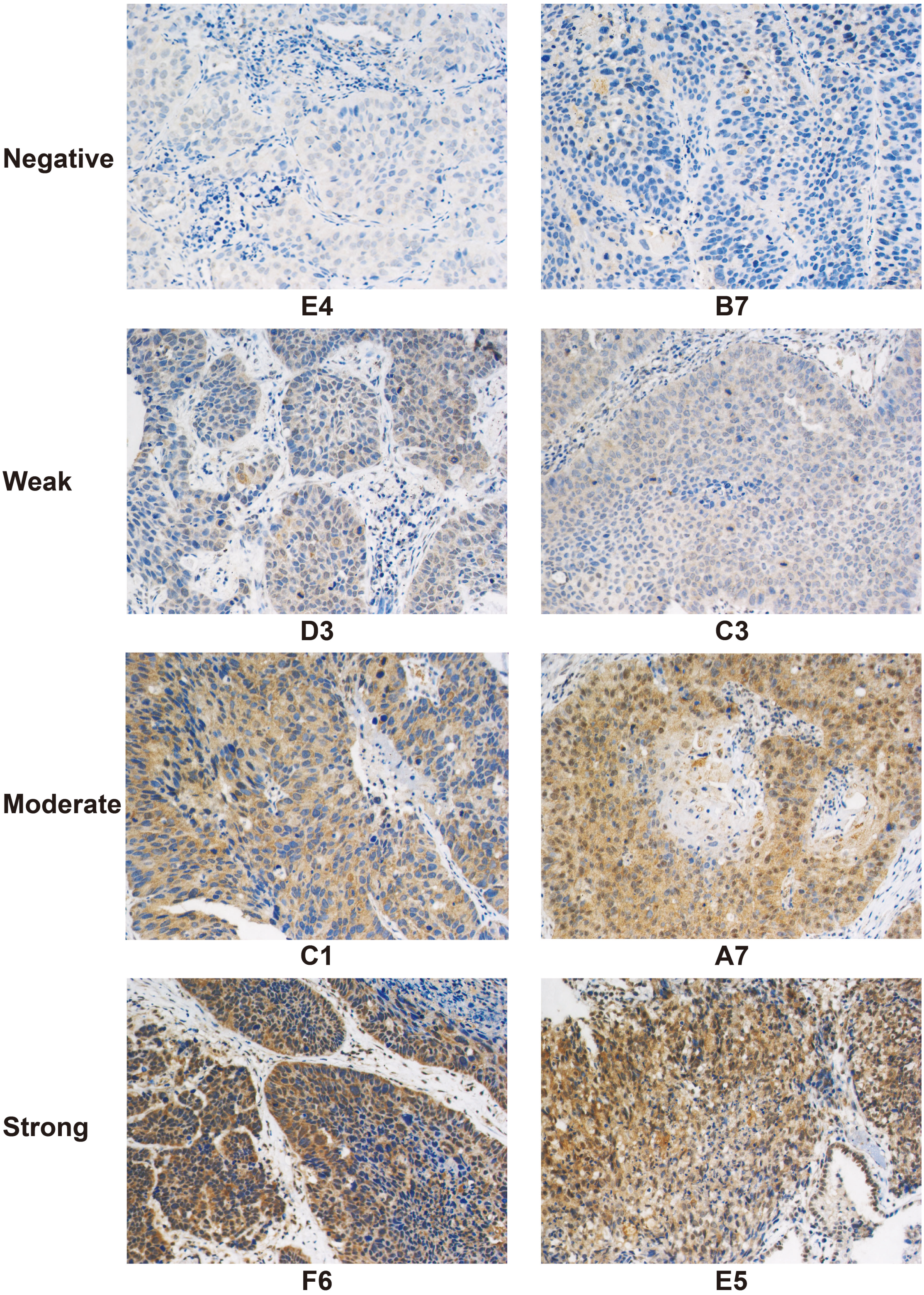

Supplementary Figure S1. Representative immunohistochemistry images of SqCLC tissue microarray cores stained for DDX56 and exhibiting different staining intensity.

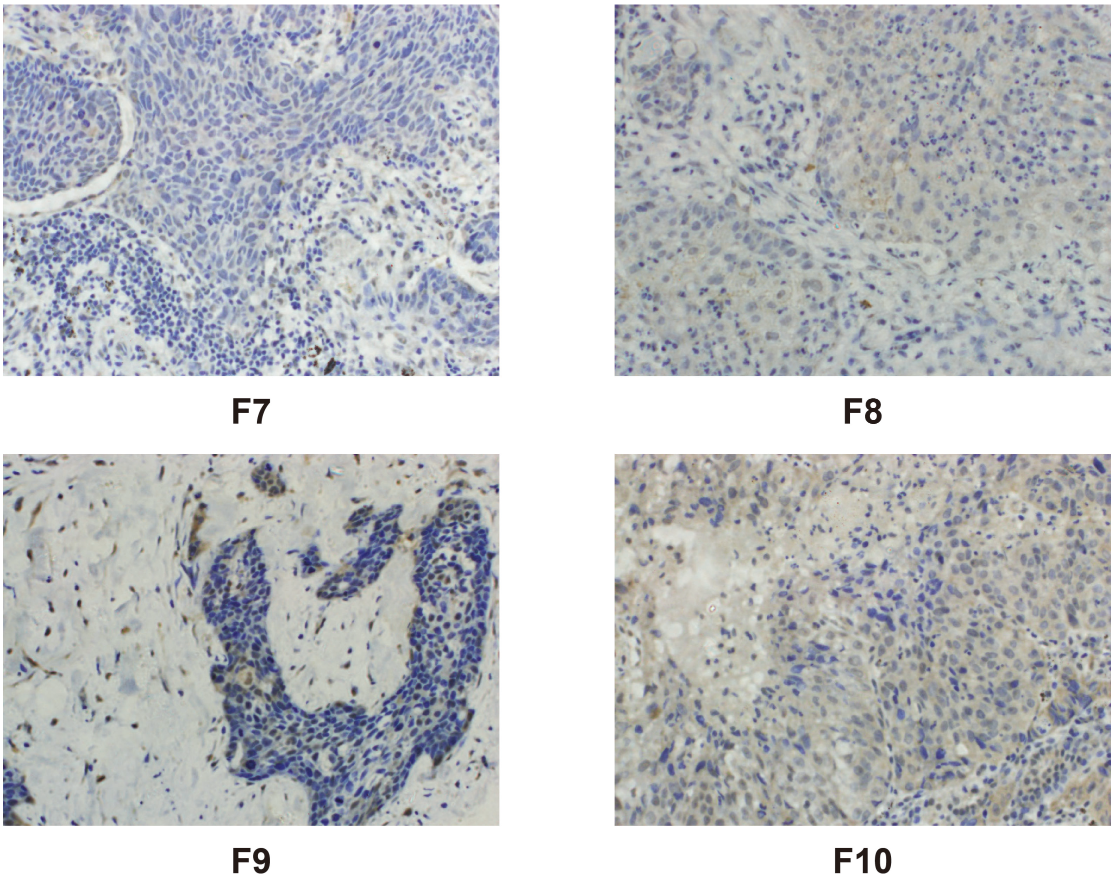

Supplementary Figure S2. Images of immunohistochemical staining for DDX56 in four cores of adjacent normal lung tissue.

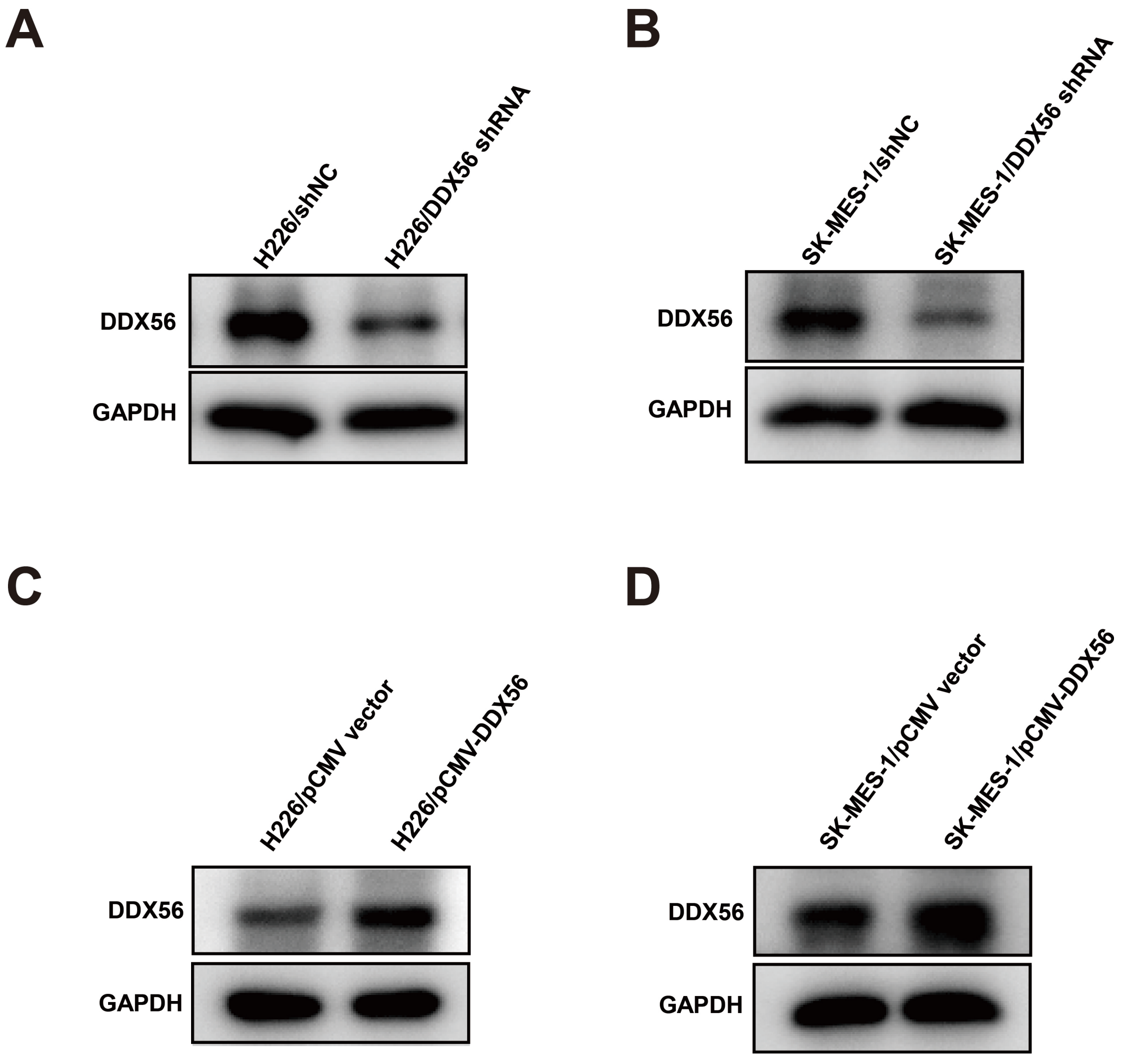

Supplementary Figure S3. Evaluation of DDX56 silencing and overexpression in SqCLC cell lines. **A** and **B**, DDX56 protein expression was determined by Western blotting 48h after transfection with DDX56 shRNA or control shRNA in H226 (**A**) and SK-MES-1 cells (**B**). **C** and **D**, DDX56 protein expression was determined by Western blotting 48h after transfection with pCMV-DDX56 vector or pCMV empty vector in H226 (**C**) and SK-MES-1 cells (**D**). GAPDH expression was used as a loading control.

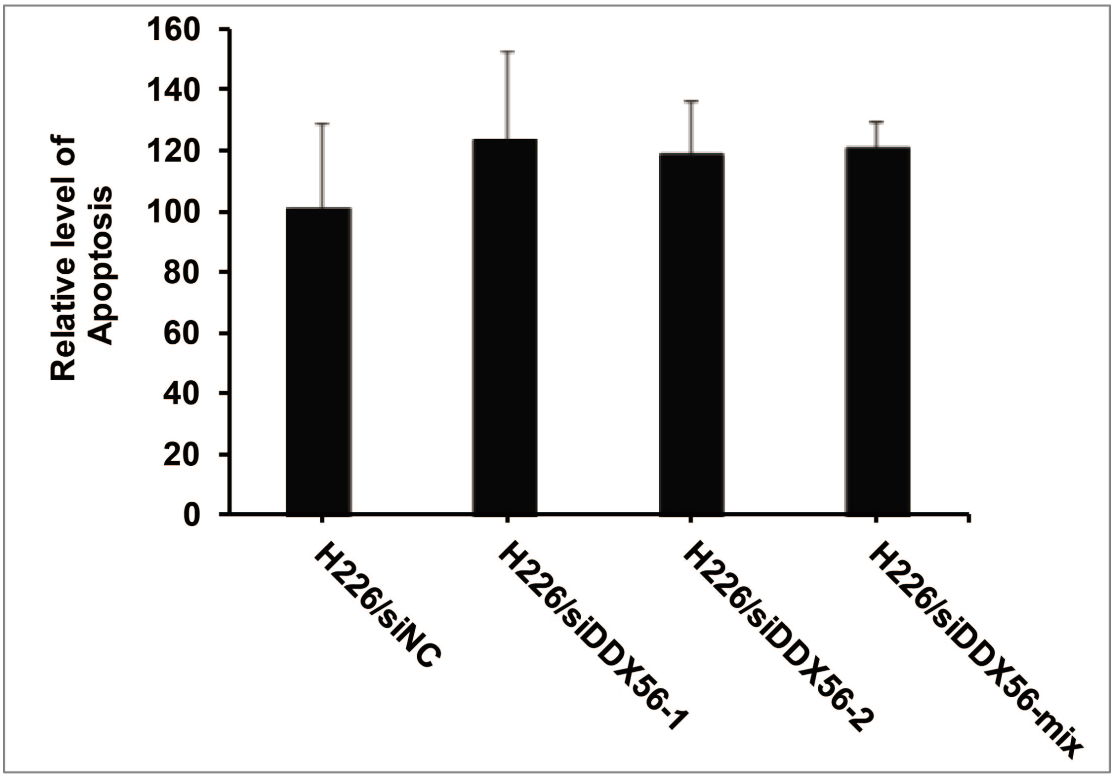

Supplementary Figure S4. Reduction of DDX56 did not significantly affect apoptosis induced by serum-free medium. H226 cells were transfected with control or DDX56 siRNAs. After overnight incubation, the cells were deprived of serum for 72 hours. Cell apoptosis was measured based on determination of cytoplasmic histone-associated DNA fragments. Data are shown as mean ± SD (n=3).

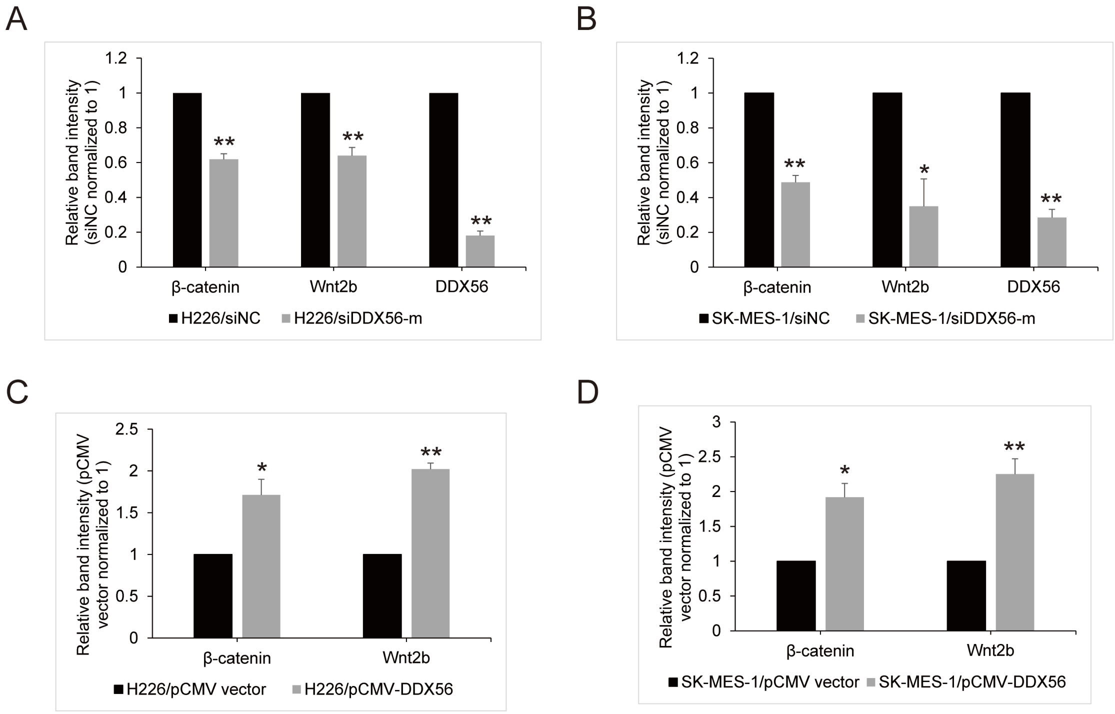

Supplementary Figure S5. Quantification analysis of Western blots shown in Figure 4. A and **B,** Quantification of Western blot analysis of β-catenin, Wnt2b, and DDX56 protein expression level in H226 and SK-MES-1 cells transfected with DDX56-specific siRNAs or control siRNA as shown in Fig. 4 D and E, confirming reduced protein expression of β-catenin and Wnt2b following DDX56 reduction in H226 (**A**) and SK-MES-1 (**B**) cells. **C** and **D**, Quantification of Western blot analysis of the expression levels of β-catenin, Wnt2b and FLAG tag-fused DDX56 protein in H226 and SK-MES-1 cells transfected with pCMV-DDX56 or control pCMV vectors as shown in Figure 4 F and G, confirming enhanced protein expression of β-catenin and Wnt2b following DDX56 overexpression in H226 (**C**) and SK-MES-1 (**D**) cells. Western blot quantification was performed using Multi Gauge software (Fujifilm). GAPDH expression was used as a loading control. Data are shown as mean ± SD (n=3, *p < 0.05; **p < 0.01).

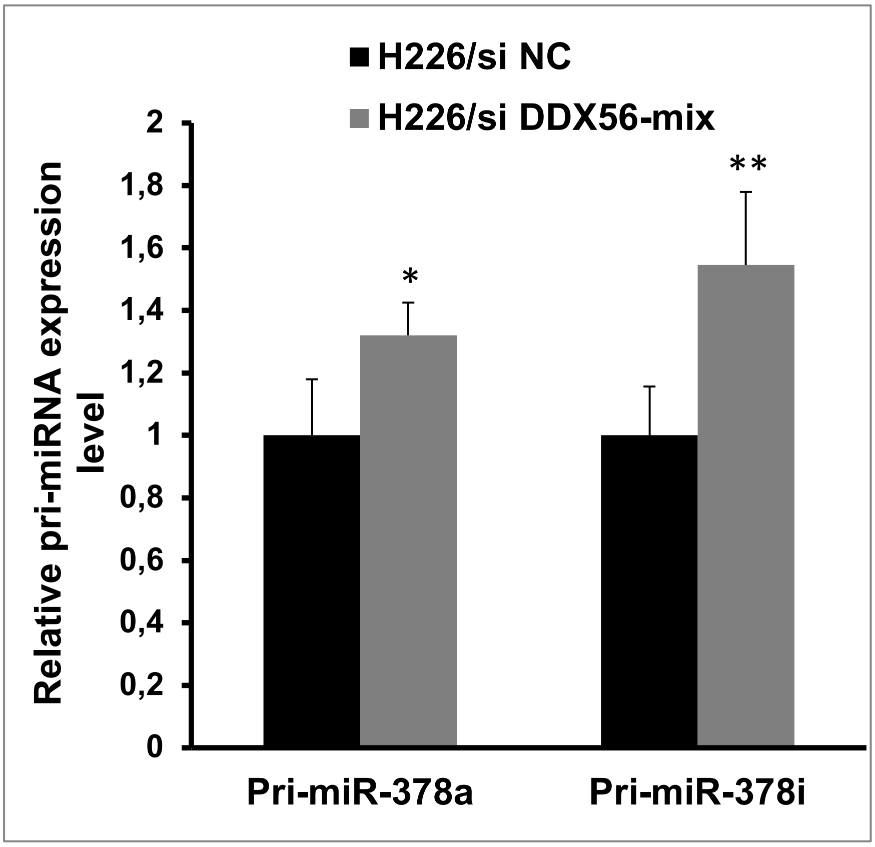

Supplementary Figure S6. qRT-PCR analysis of primary miRNA levels of miR-378a-3p and miR-378i in H226 cells transfected with DDX56-specific siRNA (si DDX56-mix) or control siRNA (si NC). GAPDH was used for normalization of the primary miRNA expression data. Data are shown as mean ± SD (n=3).

Supplementary Tables

Table S1. The clinical information of the 20 paired patients in cohort 1.

Sample index	Sex	Age	Smoke	TNM	Stage	Recurrence Time (month)	Metastasis Site	Dead time (month)
ER1	Male	68	No	T3N2M0	IIIa	5	brain	13
LR1	Male	67	No	T3N2M0	IIIa	42	lung	50
ER2	Male	71	Yes	T2bN1M0	IIb	5	brain	13
LR2	Male	72	Yes	T2bN1M0	IIb	50	brain	66
ER3	Male	63	Yes	T2bN1M0	IIb	9	brain	13
LR3	Male	61	Yes	T2bN1M0	IIb	40	bone	52
ER4	Male	64	Yes	T3N1M0	IIIa	10	Mediastinum	19
LR4	Male	62	Yes	T3N1M0	IIIa	46	lung	46
ER5	Male	67	Yes	T3N2M0	IIIa	9	Mediastinum	17
LR5	Male	68	Yes	T3N2M0	IIIa	55	Mediastinum	72
ER6	Male	72	Yes	T2aN2M0	IIIa	10	lung	19
LR6	Male	75	Yes	T2aN2M0	IIIa	36	brain	58
ER7	Male	63	Yes	T3N1M0	IIIa	10	bone	23
LR7	Male	66	Yes	T3N1M0	IIIa	39	bone	52
ER8	Male	62	Yes	T2bN1M0	IIb	9	lung	25
LR8	Male	60	Yes	T2bN1M0	IIb	35	Mediastinum	49
ER9	Male	59	Yes	T2aN2M0	IIIa	7	bone	24
LR9	Male	60	Yes	T2aN2M0	IIIa	36	Mediastinum	42
ER10	Male	66	Yes	T3N2M0	IIIa	10	lung	21
LR10	Male	63	Yes	T3N2M0	IIIa	46	bone	60

Table S3. Primers used for quantitative RT-PCR and cloning.

**Quantitative RT-PCR primers**		
**Name**	**Forward Primer (5′to 3′)**	**Reverse Primer (5′to 3′)**
DDX56	CCCATCTCGCATATTAAGCCAC	TGTACGTCCTCGTTAAAAGTAGC
CTNNB1	AAAGCGGCTGTTAGTCACTGG	CGAGTCATTGCATACTGTCCAT
GAPDH	ACCACAGTCCATGCCATCAC	TCCACCACCCTGTTGCTGTA
FZD7	CTCCCTGTGGAAAGGCATAA	CCTCTGGCTTAACGGTGTGT
WNT2B	ATTTCCCGCTCTGGAGATTT	GGTACCCTTCCTCTTGCACA
18s rRNA	CAGCCACCCGAGATTGAGCA	TAGTAGCGACGGGCGGTGTG
Pri-miR-378a-3p	ATCTTTGCCGGCCCAACTT	ACCACCAGGCCATTGGATTC
Pri-miR-378i	GTCCAGTGCTCCCTTCCAAA	AGCTGCTCCCTCCTCTTACA
miR-378a-3p	ACTGGACTTGGAGTCAGAAGGC	miScript Universal Primer
miR-378i	ACTGGACTAGGAGTCAGAAGG	miScript Universal Primer
RNU6B	CTCGCTTCGGCAGCACA	miScript Universal Primer
**Cloning primers**		
**Name**	**Forward Primer (5′to 3′)**	**Reverse Primer (5′to 3′)**
DDX56	CGCGGATCCATGGAGGACTCTGAAGCACTGGGCTTC	CCGGAATTCGGAGGGCTTGGCTGTGGGTCTG

Table S5. List of the top-10 up-regulated and down-regulated proteins in tumor tissues form ER patients vs. LR patients.

Protein name	Gene symbol	Number of paired samples showed >1.5 fold change	P-value	Protein class in The Human Protein Atlas
Top-10 up-regulated proteins in ER vs LR lung SCC patients				
Protein NDRG1	NDRG1	8	0.0059	Cancer-related genes, Disease related genes, Plasma proteins, Predicted intracellular proteins
Isoform 2 of Collagen alpha-1(VII) chain	COL7A1	7	0.002	Disease related genes, Predicted intracellular proteins, Predicted secreted proteins
Methionine aminopeptidase 1	METAP1	7	0.0098	Enzymes, Plasma proteins, Predicted intracellular proteins
Non-histone chromosomal protein HMG-17	HMGN2	7	0.0371	Predicted intracellular proteins
Phosphoserine aminotransferase	PSAT1	6	0.002	Disease related genes, Enzymes, Plasma proteins, Potential drug targets, Predicted intracellular proteins
Prefoldin subunit 3	VBP1	6	0.0039	Predicted intracellular proteins
Mitochondrial glutamate carrier 1	SLC25A22	6	0.0059	Disease related genes, Plasma proteins, Potential drug targets, Predicted membrane proteins, Transporters
Isoform 2 of Pescadillo homolog	PES1	6	0.0098	Predicted intracellular proteins
Probable ATP-dependent RNA helicase DDX56	DDX56	6	0.0156	Enzymes, Predicted intracellular proteins
CD9 antigen	CD9	6	0.0195	Cancer-related genes, CD markers, Plasma proteins, Predicted membrane proteins, Transporters
Top-10 down-regulated proteins in ER vs LR lung SCC patients				
HLA class II histocompatibility antigen, DP alpha 1 chain	HLA-DPA1	9	0.0039	Predicted intracellular proteins, Predicted membrane proteins, Predicted secreted proteins
HLA class II histocompatibility antigen, DQ alpha 1 chain (Fragment)	HLA-DQA1	8	0.0059	Predicted membrane proteins, Predicted secreted proteins
Isoform 2 of Ig mu chain C region	IGHM	7	0.0039	NA
Alcohol dehydrogenase 1B	ADH1B	7	0.0059	Enzymes, FDA approved drug targets, Plasma proteins, Predicted intracellular proteins
HLA class II histocompatibility antigen, DR alpha chain	HLA-DRA	7	0.0059	Plasma proteins, Predicted membrane proteins

MHC class II antigen	HLA-DQA1/DRA	7	0.0059	Predicted membrane proteins, Predicted secreted proteins
Immunoglobulin J chain	IGJ	7	0.0098	Plasma proteins, Predicted secreted proteins
Fructose-1,6-bisphosphatase 1	FBP1	7	0.0137	Disease related genes, Enzymes, Plasma proteins, Potential drug targets, Predicted intracellular proteins
Ig lambda chain V-III region SH	IGLV3-19	6	0.0059	NA
Cathepsin S	CTSS	6	0.0059	Enzymes, Plasma proteins, Predicted secreted proteins

Table S6. Cox univariate analysis showing correlations between selected clinical parameters and DDX56 IHC staining with overall survival in an independent SqCLC patient cohort (n=56).

Variable	OS Univariate Analysis HR (95% CI)	*p*
Age (≥60/<60 years)	1.15 (0.56-2.37)	0.696
Sex (female / male)	0.81 (0.19-3.42)	0.778
Stage (IIIa / IIb / IIa / Ib/ Ia)	1.20 (0.90-1.60)	0.206
Tumor Size (>3cm)	1.03 (0.48-2.21)	0.945
Lymphovascular invasion (yes/no)	0.97 (0.49-1.90)	0.918
Tumor location (central type / peripheral type)	1.61 (0.77-3.39)	0.208
Smoke (yes/no)	0.71 (0.27-1.89)	0.493
DDX56 staining (strong or moderate/ weak or negative)	2.28(1.03-5.02)	0.041

Table S7. Multivariate analysis showed that SqCLC patients (n=130) with high DDX56 tumor expression exhibited poor overall survival (OS) after adjustment for selected clinicopathologic characteristics.

Variable	OS Univariate Analysis HR (95% CI)	*p*	OS Multivariate Analysis HR (95% CI)	*p*
Age (⩾60/<60 years)	0.93 (0.54-1.60)	0.79	-	-
Sex (female / male)	0.79 (0.48-1.31)	0.36	-	-
Stage (IIIb/ IIIa / IIb / IIa / Ib/ Ia)	1.12(0.96-1.30)	0.17	-	-
Tumor Size (>3cm)	1.18 (0.66-2.10)	0.58	-	-
Lymph node status (2/1/0)	1.43 (1.02-2.00)	0.036	1.33 (0.94-1.88)	0.10
Smoke (yes/no)	0.70 (0.17-2.88)	0.62	-	-
DDX56 expression (⩾750/<750)	2.18 (1.34–3.55)	0.0017	2.06 (1.26–3.37)	0.0040

Table S8. Multivariate analysis showed that lung cancer patients whose tumors overexpressed high DDX56 gene expression level exhibited poor overall survival (OS) (n=1925) and progression-free survival (PFS) (n=982) after adjustment for selected clinicopathologic characteristics.

.

**Variable**	**OS Multivariate Analysis HR (95% CI)**	***p***	**RFS Multivariate Analysis HR (95% CI)**	***p***
Grade	0.99 (0.81 - 1.2)	0.8876	0.94 (0.75 - 1.19)	0.6159
AJCC stage T	1.38 (1.14 - 1.67)	0.0009	1.57 (1.23 - 2.01)	0.0003
AJCC stage N	1.83 (1.56 - 2.15)	>1E-05	1.61 (1.32 - 1.97)	>1E-05
Gender	1.12 (0.86 - 1.44)	0.4032	1.08 (0.79 - 1.48)	0.6177
Smoking history	0.79 (0.51 - 1.23)	0.2944	0.84 (0.54 - 1.33)	0.4628
DDX56 gene expression	1.53 (1.12 - 2.08)	0.0071	1.45 (1.07 - 1.98)	0.0174

Table S9. Top 10 overrepresented KEGG pathways among up- or down-regulated mRNAs following DDX56 silencing in mRNA microarray experiment.

**Overrepresented KEGG pathways**	**Count**	**P-value**	**P-value adjusted by Benjamini**
Up-regulated mRNAs			
Ribosome	66	7.3E-40	1.4E-37
Proteasome	14	6.5E-03	4.6E-01
Parkinson's disease	28	8.5E-03	4.1E-01
Huntington's disease	35	1.9E-02	5.9E-01
Oxidative phosphorylation	27	1.9E-02	5.1E-01
Spliceosome	26	2.3E-02	5.2E-01
Alzheimer's disease	31	3.6E-02	6.2E-01
tRNA biosynthesis	11	3.8E-02	5.9E-01
Adherens junction	17	4.2E-02	5.9E-01
Tight junction	26	4.6E-02	5.8E-01
Down-regulated mRNAs			
Cell cycle	44	9.1E-07	1.7E-04
Ubiquitin mediated proteolysis	45	5.5E-06	5.2E-04
Pathways in cancer	82	9.1E-05	5.8E-03
Renal cell carcinoma	25	2.5E-04	1.2E-02
Wnt signaling pathway	43	3.6E-04	1.4E-02
TGF-beta signaling pathway	28	6.6E-04	2.1E-02
Colorectal cancer	26	2.0E-03	5.3E-02
Adherens junction	24	2.8E-03	6.5E-02
Prostate cancer	26	4.7E-03	9.5E-02
Pancreatic cancer	22	5.7E-03	1.0E-01

Table S10. Summary of differentially expressed miRNAs in microarray experiment. The differentially expressed miRNAs were filtered by a fold change cutoff of 1.2 and 0.83. *=Normalized and log2-transformed intensity data

Systematic Name	H226/siDDX56-mix*	H226/siNC*	Fold Change (siDDX56 vs. siNC)	Sequence
Up-regulated miRNAs				
hsa-miR-6510-5p	3.51	3.09	1.34	CAGCAGGGGAGAGAGAGGAGTC
hsa-miR-378i	6.44	6.08	1.29	ACTGGACTAGGAGTCAGAAGG
hsa-miR-28-5p	3.84	3.49	1.27	AAGGAGCTCACAGTCTATTGAG
hsa-miR-1972	3.01	2.68	1.26	TCAGGCCAGGCACAGTGGCTCA
hsa-miR-34c-5p	3.47	3.14	1.25	AGGCAGTGTAGTTAGCTGATTGC
hsa-miR-6785-5p	5.05	4.73	1.25	TGGGAGGGCGTGGATGATGGTG
hsa-miR-22-5p	3.57	3.26	1.24	AGTTCTTCAGTGGCAAGCTTTA
hsa-miR-630	3.52	3.21	1.24	AGTATTCTGTACCAGGGAAGGT
hsa-miR-1973	4.82	4.51	1.24	ACCGTGCAAAGGTAGCATA
hsa-miR-1246	5.49	5.18	1.23	AATGGATTTTTGGAGCAGG
hsa-miR-7847-3p	3.33	3.03	1.23	CGTGGAGGACGAGGAGGAGGC
hsa-miR-197-3p	3.82	3.54	1.21	TTCACCACCTTCTCCACCCAGC
hsa-miR-378a-3p	6.51	6.24	1.21	ACTGGACTTGGAGTCAGAAGGC
hsa-miR-574-3p	3.46	3.19	1.21	CACGCTCATGCACACACCCACA
hsa-miR-26b-5p	4.8	4.54	1.2	TTCAAGTAATTCAGGATAGGT
hsa-miR-152-3p	3.17	2.9	1.2	TCAGTGCATGACAGAACTTGG
hsa-miR-31-5p	3.8	3.53	1.2	AGGCAAGATGCTGGCATAGCT
Down-regulated miRNAs				
hsa-miR-939-5p	4.4	4.92	0.7	TGGGGAGCTGAGGCTCTGGGGGTG
hsa-miR-5100	10.64	11.01	0.77	TTCAGATCCCAGCGGTGCCTCT
hsa-miR-4433a-5p	3.17	3.52	0.78	CGTCCCACCCCCCACTCCTGT
hsa-miR-1268b	4.17	4.52	0.78	CGGGCGTGGTGGTGGGGGTG
hsa-miR-3162-3p	3.3	3.64	0.79	TCCCTACCCCTCCACTCCCCA
hsa-miR-7114-5p	5.53	5.81	0.82	TCTGTGGAGTGGGGTGCCTGT
hsa-miR-6826-5p	7.81	8.09	0.82	TCAATAGGAAAGAGGTGGGACCT
hsa-miR-3659	5.68	5.96	0.83	TGAGTGTTGTCTACGAGGGCA

Table S11. Top 10 overrepresented KEGG pathways among predicted targets of up- or down-regulated miRNAs following DDX56 silencing in miRNA microarray experiment.

**Overrepresented KEGG pathways**	**Count**	**P-value**	**P-value adjusted by Benjamini**
Predicted targets of up-regulated miRNAs			
Axon guidance	36	1.8E-08	5.0E-06
Pathways in cancer	74	1.1E-07	1.6E-05
Phosphatidylinositol signaling system	27	2.8E-06	2.5E-04
MAPK signaling pathway	49	1.3E-05	8.9E-04
Focal adhesion	42	1.4E-05	7.8E-04
Wnt signaling pathway	31	3.6E-05	1.7E-03
Proteoglycans in cancer	40	3.7E-05	1.5E-03
Glioma	19	5.1E-05	1.7E-03
Signaling pathways regulating pluripotency of stem cells	30	1.2E-04	3.7E-03
Melanoma	19	1.8E-04	4.8E-03
Predicted targets of down-regulated miRNAs			
None			

References:

1. Wisniewski JR, Ostasiewicz P, Mann M: High Recovery FASP Applied to the Proteomic Analysis of Microdissected Formalin Fixed Paraffin Embedded Cancer Tissues Retrieves Known Colon Cancer Markers**.** J Proteome Res. 2011;10(7)**:**3040-9.
2. Abdallah C, Sergeant K, Guillier C, Dumas-Gaudot E, Leclercq CC, Renaut J: Optimization of iTRAQ labelling coupled to OFFGEL fractionation as a proteomic workflow to the analysis of microsomal proteins of Medicago truncatula roots**.** Proteome Sci. 2012;10(1):37.
3. Sheng Q, Dai J, Wu Y, Tang H, Zeng R: BuildSummary: using a group-based approach to improve the sensitivity of peptide/protein identification in shotgun proteomics**.** J Proteome Res. 2012;11(13)**:**1494-1502.
